# Should we screen for the sexually-transmitted infection *Mycoplasma genitalium*? Evidence synthesis using a transmission-dynamic model

**DOI:** 10.1038/s41598-017-16302-8

**Published:** 2017-11-23

**Authors:** Ruthie Birger, John Saunders, Claudia Estcourt, Andrew John Sutton, Catherine H. Mercer, Tracy Roberts, Peter J. White

**Affiliations:** 1 MRC Centre for Outbreak Analysis & Modelling, Department of Infectious Disease Epidemiology, School of Public Health, Imperial College London, Norfolk Place, London, W2 1PG UK; 2 Earth Institute & Department of Environmental Health Sciences, Mailman School of Public Health, Columbia University, 722 W 168th Street, New York, NY USA; 3 Centre for Immunology & Infectious Disease, Blizard Institute, Barts and The London School of Medicine & Dentistry, The Blizard Building, 4 Newark Street, London, E1 2AT UK; 4 Barts Health NHS Trust, Turner St, Whitechapel, London, E1 1BB UK; 5 School of Health and Life Sciences, Glasgow Caledonian University, Cowcaddens Road, Glasgow, G4 0BA Scotland UK; 6 Health Economics Unit, Leeds Institute of Health Sciences, University of Leeds, Level 11, Worsley Building, Clarendon Way, Leeds, LS2 9JT UK; 7 NIHR Diagnostic Evidence Co-operative, Clinical Sciences Building, St James’s University Hospital, Leeds, LS9 7TF UK; 8 Institute for Global Health, University College London, 3rd floor Mortimer Market Centre off Capper Street, London, WC1E 6JB, UK; 9 Health Economics Unit, University of Birmingham, Edgbaston, Birmingham, B15 2TT UK; 10 NIHR Health Protection Research Unit in Modelling Methodology, Department of Infectious Disease Epidemiology, School of Public Health, Imperial College London, Norfolk Place, London, W2 1PG UK; 11 Modelling & Economics Unit, National Infection Service, Public Health England, 61 Colindale Avenue, London, NW9 5EQ UK

## Abstract

There is increasing concern about *Mycoplasma genitalium* as a cause of urethritis, cervicitis, pelvic inflammatory disease (PID), infertility and ectopic pregnancy. Commercial nucleic acid amplification tests (NAATs) are becoming available, and their use in screening for *M*. *genitalium* has been advocated, but *M*. *genitalium*’s natural history is poorly-understood, making screening’s effectiveness unclear. We used a transmission-dynamic compartmental model to synthesise evidence from surveillance data and epidemiological and behavioural studies to better understand *M*. *genitalium’s* natural history, and then examined the effects of implementing NAAT testing. Introducing NAAT testing initially increases diagnoses, by finding a larger proportion of infections; subsequently the diagnosis rate falls, due to reduced incidence. Testing only symptomatic patients finds relatively little infection in women, as a large proportion is asymptomatic. Testing both symptomatic and asymptomatic patients has a much larger impact and reduces cumulative PID incidence in women due to *M*. *genitalium* by 31.1% (95% range:13.0%-52.0%) over 20 years. However, there is important uncertainty in *M*. *genitalium’s* natural history parameters, leading to uncertainty in the absolute reduction in PID and sequelae. Empirical work is required to improve understanding of key aspects of *M*. *genitalium*’s natural history before it will be possible to determine the effectiveness of screening.

## Introduction

There is increasing concern about the sexually-transmitted bacterium, *Mycoplasma genitalium*, as a cause of a range of morbidities^1,2^ including mucopurulent cervicitis^1,3–6^, pelvic inflammatory disease (PID)^1,5–11^, tubal factor infertility^1,12,13^, and ectopic pregnancy in women^7–10,12–18^; urethritis, balanitis, posthitis and balanoposthitis in men^19–21^; and increased HIV acquisition and transmission^22^. An increasing array of commercial nucleic acid amplification tests (NAATs) are available, for example, the Bio-Rad Dx CT/NG/MG® assay^23^, many of which are non-invasive (urine-based), and using them to screen for *M*. *genitalium* has been advocated^24^, and this may reduce the incidence of serious, costly sequelae in women^25^.

However, there is uncertainty in its importance as a cause of disease, whether public health intervention is justified and, if so, how best to intervene^14,25–27^. Uncertainty in the natural history of *M*. *genitalium* needs to be accounted for in analyses of the likely impact of screening^26^. Extensive screening for *Chlamydia trachomatis* has been implemented by several countries – for more than a decade in England, and yet the impact of this screening is still poorly-understood^28^. In large part this is because natural history of *C*. *trachomatis* is still not well-understood, reflected by wide variation in the values of key parameters used in mathematical models^29^; only in recent years has evidence synthesis been used to improve estimates^30,31^.

As highlighted by Walker *et al*. “transmission dynamics and duration of infection [are] both important factors in understanding the management of [*M*. *genitalium*] in the population”^14^. We used a transmission-dynamic model to synthesise evidence^32^ from studies of the natural history and epidemiology of *M*. *genitalium*, and surveillance data, to identify the parameters that are most important in contributing to uncertainty in its transmission dynamics. We then examined the potential impact of using NAATs for (i) diagnostic testing of symptomatic patients, and (ii) diagnostic testing of symptomatic patients plus screening of asymptomatic patients, of both sexes, on incidence of infection and diagnoses in each sex and incidence of serious sequelae in women.

## Methods

### Model structure

**Figure 1:**
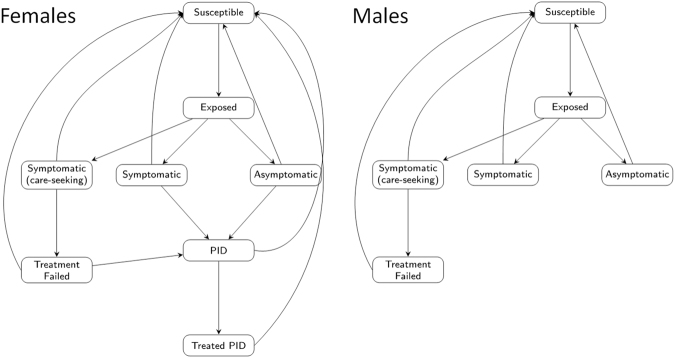
Model flow diagram illustrating schematically the natural history of *M*. *genitalium* infection and the care-seeking behaviour of infected individuals. Compartments represent mutually exclusive states of individuals, and arrows indicate the direction of flow between compartments. Susceptible individuals are uninfected. All other compartments contain infected individuals, all of whom are infectious except for those with latent infection. Upon progression from latent infection, all individuals have signs of disease and symptomatic individuals also have symptoms. Some individuals with symptoms seek care due to their symptoms; others do not. Arrows showing return to the uninfected Susceptible state indicate both natural recovery and treatment. The model population is stratified into males and females and into classes with low, medium, and high rates of sexual partner change (not shown). Only females can experience PID.

We developed a transmission-dynamic compartmental mathematical model in which *M*. *genitalium*’s epidemiology is represented by compartments for asymptomatic infected individuals, symptomatic infected individuals not seeking treatment, symptomatic infected individuals seeking treatment, and women with asymptomatic, symptomatic and treated PID^33^. The model is stratified by sex, and into ‘activity classes’ with low, medium, and high rates of sexual partner change. Interaction between activity classes is determined by a mixing matrix, with an assortativeness coefficient specifying the amount of like-with-like interaction^34^. The model is summarised by the flow diagram in Fig. 1; equations specifying the model are in the Appendix. Acquisition of infection causes individuals to move from the Susceptible state to the Latent state, from which they progress to one of the Infectious states. Infectious individuals may be Asymptomatic, Symptomatic but not seeking care despite their symptoms, or Symptomatic and seeking care. Individuals may seek care following partner notification: this applies to persons with and without infection and with and without symptoms. When an infected individual is diagnosed and treated successfully, they return to the Susceptible state. If treatment is unsuccessful, then the patient enters the Treatment Failure state; those individuals then seek further treatment due to continuing symptoms (this time treated successfully) or recover through natural immune processes. Infected individuals who do not receive treatment will recover eventually through natural immune processes, returning to the Susceptible state. If an Asymptomatic individual is treated and treatment fails, then they remain in the Asymptomatic infected state, and recover naturally – unless partner notification leads to their being diagnosed and treated again. In women, a proportion of cases of untreated infection and of treatment failure cases will progress to PID. A proportion of PID cases are symptomatic and seek care due, entering the Treated PID state, with successful treatment returning them to the Susceptible state. All cases of PID can recover through natural immune processes and return to the Susceptible state. Numbers of cases of ectopic pregnancy and tubal factor infertility were calculated by multiplying the number of untreated PID cases by the proportions of PID cases developing each sequela as reported in previous studies^15–17,30^.

We used surveillance data from England, where all symptomatic men and 5% of asymptomatic men attending sexual health clinics received screening for non-chlamydial non-gonococcal urethritis (NCNGU) with urethral gram stain microscopy^35^ and diagnosed cases were recorded. In general practice, microscopy is not used at all and people are managed according to symptoms. Women with symptoms of mucopurulent cervicitis would be treated (and the diagnosis recorded in surveillance), whilst asymptomatic women with infection would only be treated (and recorded in surveillance) through partner notification.

Symptomatic cases of NCNGU were given first-line treatment with azithromycin; a proportion of these cases (estimates are described below) will have been due to *M*. *genitalium*, and a proportion of those will have been cured by it. *M*. *genitalium* can be treated with azithromycin, doxycycline and cefoxitin, with treatment failure rates ranging from 5–60%^9,36–39^. Moxifloxacin is more effective but is often reserved as a second-line treatment^38^.

### Sensitivity analysis to identify the most epidemiologically important parameters

To identify which parameters have the greatest impact on the model output (i.e. diagnoses in men and prevalence in women), prior ranges for parameters values were defined, based on literature and expert opinion (Table 1)^8,9,36,40–44^. Some parameter estimates were well-defined, including sexual partner change rates^45^; time-delays associated with seeking and receiving care^46,47^; the proportion of symptomatic patients abstaining from sexual activity whilst seeking care^46–48^; and the effectiveness of treatment^41,49^. Natural history parameters of *M*. *genitalium* were more uncertain, due to variation between studies of estimates or due to a lack of studies. These include the transmission probability; the proportion of infections that are symptomatic; the latent period and duration of infection; and the rate of progression to PID. In the univariate analysis, the model was run using intermediate values of all the ranges of the parameters while allowing one parameter at a time to vary between the minimum and maximum of the range. The most influential parameters were then varied in the model calibration step.

**Table 1:** Model parameters. Parameters which were sampled have prior and posterior ranges reported; other parameter values were fixed. “EO” denotes expert opinion. Adapted from Estcourt *et al.* 2016.

**Description**	**Symbol**	**Prior range**			**Posterior range**			**Source**
		**Low**	**Mean**	**High**	**Low**	**Mean**	**High**	
Rate of aging in and out (per year)	*μ*		1/22					22-yr age range
Proportion of population in low risk activity class	*p*_*low*_		0.56					55,64
Proportion of population in medium risk activity class	*p*_*med*_		0.3					55,64
Proportion of population in high risk activity class	*p*_*high*_		0.14					55,64
Partner change rate in low risk activity class (per year)	*r*_*low*_		0.15					55,64
Partner change rate in medium risk activity class (per year)	*r*_*med*_		0.76					55,64
Partner change rate in high risk activity class (per year)	*r*_*high*_		8.49					55,64
Sex-act frequency (per 4 weeks)	*q*		7					65
Sexual mixing assortativeness coefficient	*ε*	0.1	0.5	0.9	0.1	0.42	0.9	EO
Per-sex act transmission probability: male to female	*ϕ(f)*	0.01	0.155	0.3	0.07	0.18	0.30	3,61,66–68
Per-sex act transmission probability: female to male	*ϕ(m)*	0.01	0.155	0.3	0.08	0.23	0.3	3,61,66–68
Latent period (days)	*d*_*lat*_		14					19
Proportion of incident infections that is symptomatic: female	*z(f)*	0.01	0.355	0.7	0.09	0.38	0.7	3,13,69,70
Proportion of incident infections that is symptomatic: male	*z(m)*	0.1	0.4	0.7	0.1	0.2	0.57	3,13,69,70
Proportion of symptomatic infecteds who seek care spontaneously: female	*ρ(f)*	0.01	0.6	0.9	0.01	0.26	0.87	EO
Proportion of symptomatic infecteds who seek care spontaneously: male	*ρ(m)*	0.6	0.795	0.99	0.6	0.77	0.99	EO
Time from symptom onset to seeking care (days)	*d*_*seek*_	0	10	20	0.05	9.46	19.76	46,47
Time from seeking care to obtaining it (days)	*d*_*care*_		7					46
Proportion of infecteds seeking care abstaining from sex: female	*α(f)*	0.2	0.4	0.6	0.2	0.4	0.6	46–48
Proportion of infecteds seeking care abstaining from sex: male	*α(m)*	0.4	0.6	0.8	0.4	0.59	0.8	46–48
Per-capita rate of sexual-health screening in those without symptoms (per day)	*σ(f)*		0.24					EO
Per-capita rate of sexual-health screening in those without symptoms, male (per day)	*σ(m)*	0.001	0.089	0.138	0.005	0.076	0.132	71
Proportion of patients who go directly to GUM	*p*_*GUMD*_	0.3	0.5	0.7	0.303	0.47	0.7	72
Proportion of patients who go to GUM from GP	*p*_*GPGUM*_	0.1	0.25	0.4	0.103	0.24	0.4	42
Proportion of men without symptoms who are screened by microscopy at GUM	*η*		0.05					35
Specificity of microscopy	*spec*_*m*_		97%					73
Sensitivity of microscopy	*sens*_*m*_		80%					73
Specificity of NAAT test	*spec*_*p*_		96%					74
Sensitivity of NAAT test	*sens*_*p*_		87%					74
Partner notification look-back period (days)	*d*_*look*_		60					75
Proportion of partners traced from index patients diagnosed in GUM	*f*_*GUM*_	0.01	0.55	0.7	0.03	0.28	0.6	50,76
Proportion of partners traced from index patients diagnosed in GP	*f*_*GP*_	0.01	0.255	0.5	0.01	0.19	0.5	42,43
Mean delay in treatment of contacts (days)	*τ*		10					EO
Treatment failure proportion (without NAAT testing)	*ζ*	0.05	0.351	0.6	0.05	0.28	0.596	9,36,38,39,41
Treatment failure proportion (second-line without NAAT testing; first line with NAAT testing)	*ζ*	—	0.05	—	—	—	—	2,27
Natural recovery rate: female (per year)	*γ(f)*	0.3	1.2	2.4	0.46	1.23	2.37	8,11,38,60,62
Natural recovery rate: male (per year)	*γ(m)*	0.3	0.65	1	0.3	0.6	0.955	11,38,62
Natural recovery rate following treatment failure (per year)	*γ*_*F*_		2					EO
Duration of treatment-failed infection before retreatment (months)	*d*_*inf2*_		1					EO
Natural recovery rate from PID (per year)	*γ*_*PID*_		0.25					EO
Rate of progression to PID – calibration (per day)	*ψ*_*P*_		0.022					11
Rate of progression to PID – scenario analysis (per day)	*ψ*_*P*_		0.022, 0.044, 0.09					6,11
Proportion of those with PID seeking treatment	*y*		0.5					EO
Mean duration of PID before treatment (days)	*d*_*PT*_		40					EO
PID treatment duration (days)	*d*_*tp*_		21					EO
Proportion of PID cases developing tubal factor infertility	*p*_*TFI*_		0.08					15–17,30
Proportion of PID cases developing ectopic pregnancy	*p*_*EP*_		0.12					15–17,30

### Model calibration

The model represents the UK population aged 18–40 years, with 10 million individuals of each sex. Since testing for *M*. *genitalium* is not routine, we use surveillance data on diagnoses of NCNGU in men from sexual health clinics in the UK in 2000–2009^50^, complemented by estimates of the proportion of NCNGU that is due to *M*. *genitalium* (i.e. 10–46%^19,20,51,52^). In men in the UK, there were ~65,000 annual diagnoses of urethritis due to NCNGU, ~10% of which were asymptomatic epidemiologically-treated cases^50^. The model was calibrated to estimated numbers of annual *M*. *genitalium* diagnoses in men (including asymptomatic epidemiologically-treated cases), and the prevalence in women (i.e. 3.3% (95% CI: 2.6–4.1%))^11^. Candidate parameter sets were generated by Latin Hypercube Sampling from the prior ranges of parameters that were uncertain and influential. Parameter sets were accepted if both the annual diagnoses and prevalence fell within the specified ranges defined by the data: the female prevalence generated by the model had to fall in the range 2.6–4.1%, and the annual number of male diagnoses had to fall between 6,500–29,900 (i.e.10–46% of 65,000 NCNGU diagnoses). The parameter-selection process was run until 200 accepted parameter sets were obtained (from ~40,000 candidate sets), which comprise the posterior distribution. Sensitivity analysis was then performed to determine the most influential parameters, by calculating partial rank correlation coefficients (PRCCs)^53^.

After selection of parameter sets to represent uncertainty in transmission dynamics, the model was used to examine the impact of NAAT testing of (i) symptomatic individuals of both sexes in both genitourinary medicine (GUM) and general practice (GP) clinics (plus the 5% of asymptomatic patients in GUM who were being tested by microscopy because those clinics would be unlikely to stop testing that patient group), and (ii) all symptomatic patients of both sexes in both GUM and GP clinics plus asymptomatic patients attending GUM. In addition to higher diagnosis rates, it was assumed that NAAT testing would allow for more effective treatment, so that treatment failure rates would be lower. In scenario analysis, we varied the progression rate parameter, $\psi$, using values of 0.022, 0.044, and 0.09, which correspond to percentages of *M*. *genitalium* infections in women progressing to PID of 2.1%, 4.5%, and 8.5%^6,11^.

Model code (available on request) was implemented using Matlab version 2016b.

## Results

### Model sensitivity analysis and calibration

The model was able to reproduce the observed epidemiological data (diagnosis rates in men and prevalence in women). In the univariate sensitivity analysis that was performed to determine which parameters would be varied in the model calibration step, the most influential parameters associated with uncertainty in the model output were proportion of infections that are symptomatic; proportion of symptomatics abstaining from sex; the proportion of those patients who seek care; time from onset to care-seeking; per-capita rate of care-seeking; proportion of patients who go to GUM, directly or via GP; sexual mixing pattern (assortativeness coefficient); transmission probabilities; proportion of partners traced; treatment failure rate; and natural recovery rates. (Table 1, parameters with prior and posterior ranges, Tables 2, 3 for post-fitting PRCC calculations as described below).

**Table 2:** Sensitivity of model to values of sampled parameters.

Annual reports of symptomatic males diagnosed in GUM			Female prevalence			Annual reports of epidemiologically-treated males		
Parameter Description	Parameter Name	PRCC	Parameter Description	Parameter Name	PRCC	Parameter Description	Parameter Name	PRCC
Proportion Symptomatic (M)	*z(m)*	0.73**	Proportion Care-seeking (F)	$\rho (f)$	−0.28*	Proportion Care-seeking (F)	$\rho (f)$	0.74**
Transmission Probability (F→M)	$\varphi (f)$	0.54**	Recovery rate (F)	$\gamma (f)$	−0.20*	Assortativeness Coefficient	$\epsilon$	0.65**
Proportion of patients who go straight to GUM	*p*_*GUMD*_	0.49**	Assortativeness Coefficient	$\epsilon$	−0.18*	Proportion Symptomatic (F)	*z(f)*	0.64**
Proportion Care-seeking (M)	$\rho (m)$	0.43**	Proportion Symptomatic (M)	*z(m)*	−0.17*	Proportion of patients who go straight to GUM	*p*_*GUMD*_	0.33**
Proportion Care-seeking (F)	$\rho (f)$	−0.22*	Transmission Probability (M→F)	$\varphi (m)$	0.15*	Proportion of patients who go to GUM from GP	*p*_*GPGUM*_	0.21*
Proportion of patients who go to GUM from GP	*p*_*GPGUM*_	0.17**	Proportion Symptomatic (F)	*z(f)*	−0.14*	Transmission Probability (F→M)	$\varphi (f)$	0.16*
Assortativeness Coefficient	$\epsilon$	−0.15*	Recovery rate (M)	$\gamma (m)$	−0.11	Recovery rate (F)	$\gamma (f)$	0.15*
Recovery rate (F)	$\gamma (f)$	−0.14*	Proportion Care-seeking (M)	$\rho (m)$	−0.10	Transmission Probability (M→F)	$\varphi (m)$	0.13
Proportion Symptomatic (F)	*z(f)*	−0.13	Proportion of patients who go straight to GUM	*p*_*GUMD*_	−0.09	Reduction in sexual activity when care-seeking (F)	*α(f)*	−0.08
Transmission Probability (M→F)	$\varphi (m)$	0.11	Proportion of patients who go to GUM from GP	*p*_*GPGUM*_	−0.07	Recovery rate (M)	$\gamma (m)$	−0.06
**p <0.001, *p<0.05								

The table presents partial rank correlation coefficients (PRCCs) for the varied parameters with respect to annual numbers of symptomatic males diagnosed in GUM, prevalence in females, and annual numbers of epidemiologically-treated males. In each case, parameters are ranked by their importance, with statistically-significant effects indicated. In the Parameter Description columns, “(F)” and “(M)” refer to female and males, respectively.

**Table 3:** Sensitivity of model to values of sampled parameters.

Female Incidence Reduction			Male Incidence Reduction			PID Prevalence Reduction		
Parameter Description	Parameter Name	PRCC	Parameter Description	Parameter Name	PRCC	Parameter Description	Parameter Name	PRCC
Care-seeking rate of Asymptomatic Males	*σ(m)*	0.67**	Care-seeking rate of Asymptomatic Males	*σ(m)*	0.60**	Care-seeking rate of Asymptomatic Males	*σ(m)*	0.67**
Proportion Symptomatic (F)	*z(f)*	−0.51**	Proportion Symptomatic (F)	*z(f)*	−0.51**	Proportion Symptomatic (F)	*z(f)*	−0.58**
Transmission Probability (M→F)	$\varphi (m)$	−0.38**	Transmission Probability (F→M)	$\varphi (f)$	−0.38**	Recovery rate (F)	$\gamma (f)$	−0.35**
Assortativeness Coefficient	$\epsilon$	0.34**	Assortativeness Coefficient	$\epsilon$	0.36**	Transmission Probability (F→M)	$\varphi (f)$	−0.32**
Recovery rate (F)	$\gamma (f)$	−0.29**	Recovery rate (F)	$\gamma (f)$	−0.32**	Assortativeness Coefficient	$\epsilon$	0.30**
Proportion of patients who go to GUM from GP	*p*_*GPGUM*_	0.16*	Proportion Care-seeking (F)	$\rho (f)$	0.16*	Proportion Care-seeking (F)	$\rho (f)$	0.17*
Proportion Care-seeking (F)	$\rho (f)$	0.14	Proportion of patients who go straight to GUM	*p*_*GUMD*_	0.14	Proportion of patients who go to GUM from GP	*p*_*GPGUM*_	0.16*
Proportion of patients who go straight to GUM	*p*_*GUMD*_	0.13	Proportion of patients who go to GUM from GP	*p*_*GPGUM*_	0.13	Proportion of patients who go straight to GUM	*p*_*GUMD*_	0.15*
Proportion Care-seeking (M)	$\rho (m)$	0.09	Proportion Care-seeking (M)	$\rho (m)$	0.09	Proportion Symptomatic (M)	$z(m)$	−0.07
**p <0.001, *p<0.05								

The table presents partial rank correlation coefficients (PRCCs) for the varied parameters with respect to annual numbers of reductions in female and male incidence and PID prevalence. In each case, parameters are ranked by their importance, with statistically-significant effects indicated. In the Parameter Description column, “(F)” and “(M)” refer to female and male, respectively.

**Figure 2:**
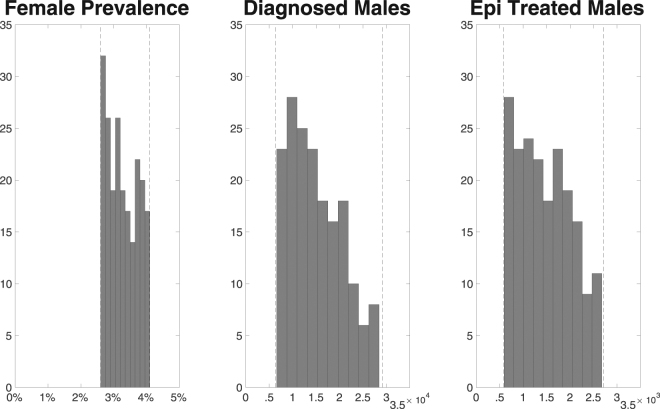
Comparison of model prevalence and diagnoses to data. Panels contain histograms showing the distributions of the prevalence in females and annual diagnoses in males produced by the sets of accepted parameter values from the calibration process.

**Figure 3:**
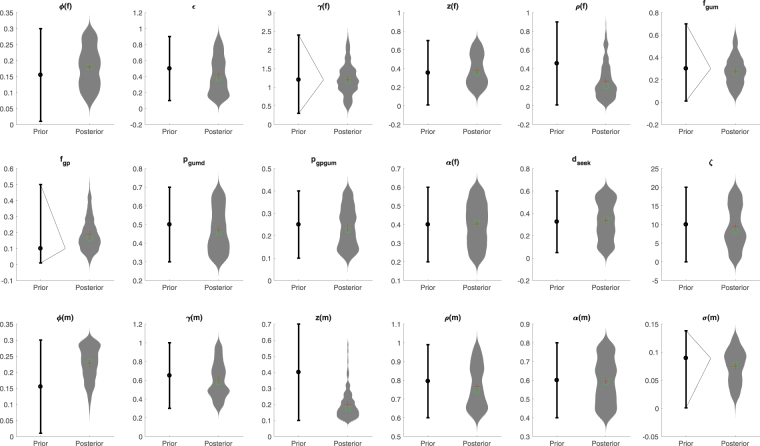
Comparison of prior and posterior parameter values. For sampled parameters, the prior and posterior distributions (min, mean, max) are shown: the prior distributions are on the left of each pair. Prior distributions were all uniform, except where triangular, as indicated on the relevant plots. Posterior distributions are shown as violin plots, with red crosses representing the mean values, and green boxes representing the medians. Parameters are as follows: *ϕ(f)*, *ϕ(m)*: Per-sex act transmission probability (male to female, male to female, respectively); ε: Sexual mixing assortativeness coefficient; *γ(f)*, *γ(m)*: Natural recovery rate (female, male, respectively); *z(f)*, *z(m)*: Proportion of incident infections that is symptomatic (female, male, respectively); *ρ(f)*, *ρ(m)*: Proportion of symptomatic infecteds who seek care spontaneously (female, male, respectively); *f*_*GUM*_: Proportion of partners traced from index patients diagnosed in GUM; *f*_*GP*_: Proportion of partners traced from index patients diagnosed in GP; *p*_*GUMD*_: Proportion of patients who go directly to GUM; p_GPGUM_: Proportion of patients who go to GUM from GP; *α(f)*, *α(m)*: Proportion of infecteds seeking care who abstain from sex (female, male, respectively); *d*_*seek*_, Time from symptom onset to seeking care; *ζ*, Treatment failure proportion (first-line treatment); *σ(m)*, Per-capita rate of sexual-health screening in those without symptoms (male).

For parameters that were varied in probabilistic sampling, the prior ranges are reported in Table 1, with the distribution of model prevalence in females and annual diagnoses in males amongst the posterior parameter sets shown in Fig. 2. Posterior parameter distributions are reported in Table 1 and plotted in Fig. 3. For some of the parameters, the prior and posterior ranges and mean values were similar (Fig. 3), indicating that the priors were in agreement with the other available data that we synthesised, but also that those data provided only limited additional information on these parameter values, indicated by the limited reduction in the range of uncertainty. For other parameters, related to sexual behaviour (*ε*, *σ(m)*), natural history of infection (*ϕ(f)*, *ϕ(m)*, *z(f)*, *z(m)*, *γ(f)*, *γ(m)*), use of and performance of the health service (*ρ(f)*, *d*_*seek*_, *f*_*GUM*_, *f*_*GP*_), and the treatment failure proportion (*ζ*), prior and posterior mean values were different, indicating that the surveillance data were informative, although in most cases the prior and posterior ranges were similar.

There is uncertainty in the baseline number of PID cases due to *M*. *genitalium*, with estimates of 155,700 (95% range 69,000–273,000), 306,000 (134,000–545,000), and 605,000 (256,000–1,120,000) over 20 years, corresponding to proportions of untreated infections in women progressing to PID of 2.1%, 4.5%, and 8.5%, respectively (Table 4 and Supplementary Tables 1 & 2), which are generated by using *ψ* values of 0.022, 0.044, and 0.09, respectively. Price *et al*.^30^ estimated the incidence of all-cause PID to be 1.8% p.a., which equates to 192,000 cases annually in a population of the size used in the model, and corresponds to 3,843,000 cases over 20 years. This means that the proportion of all-cause PID that is estimated to be due to *M*. *genitalium* is 4.1%, 8.0%, or 15.7%, corresponding to proportions of untreated infections in women progressing to PID of 2.1%, 4.5%, and 8.5%, respectively. For comparison, Price *et al*.^30^ estimated that 14.8% of chlamydia infections progress to PID, and that 19.7% of all-cause PID is due to chlamydia.

**Table 4:** Numbers of cases of serious sequelae in women due to *M*. *genitalium* in the different scenarios, over 20 years.

**Cumulative incidence**	**Mean**	**Weighted mean**	**Median**	**95% range**	**Difference from baseline**	
					**Mean**	**95% range**
***Pelvic Inflammatory Disease***						
Baseline	309,000	306,000	293,000	134,000–545,000	—	—
NAAT testing for symptomatic patients	275,000	272,000	259,000	125,000–519,000	33,800	~0–87,800
NAAT testing for all patients	219,000	216,000	205,000	75,900–458,000	89,600	40,400–150,000
***Tubal Factor Infertility***						
Baseline	18,500	18,400	17,600	8,000–32,700	—	—
NAAT testing for symptomatic patients	16,500	16,300	15,500	7,500–31,100	2,000	~0–5,300
NAAT testing for all patients	13,100	13,000	12,300	4,600–27,500	5,400	2,400–9,000
***Ectopic Pregnancy***						
Baseline	12,400	12,300	11,700	5,400–21,800	—	—
NAAT testing for symptomatic patients	11,000	10,900	10,300	5,000–20,800	1,400	~0–3,500
NAAT testing for all patients	8,800	8,600	8,200	3,000–18,300	3,600	1,600–6,000

In the scenarios presented here 4.5% of infections progress to PID. Weighted mean figures are averages of results obtained using the accepted parameter sets, weighted by the likelihood of each parameter set. The Difference columns report the difference between the baseline and intervention scenarios, calculated using the likelihood-weighted mean estimates, and the 95% ranges of the differences.

### Impact of NAAT testing

**Figure 4:**
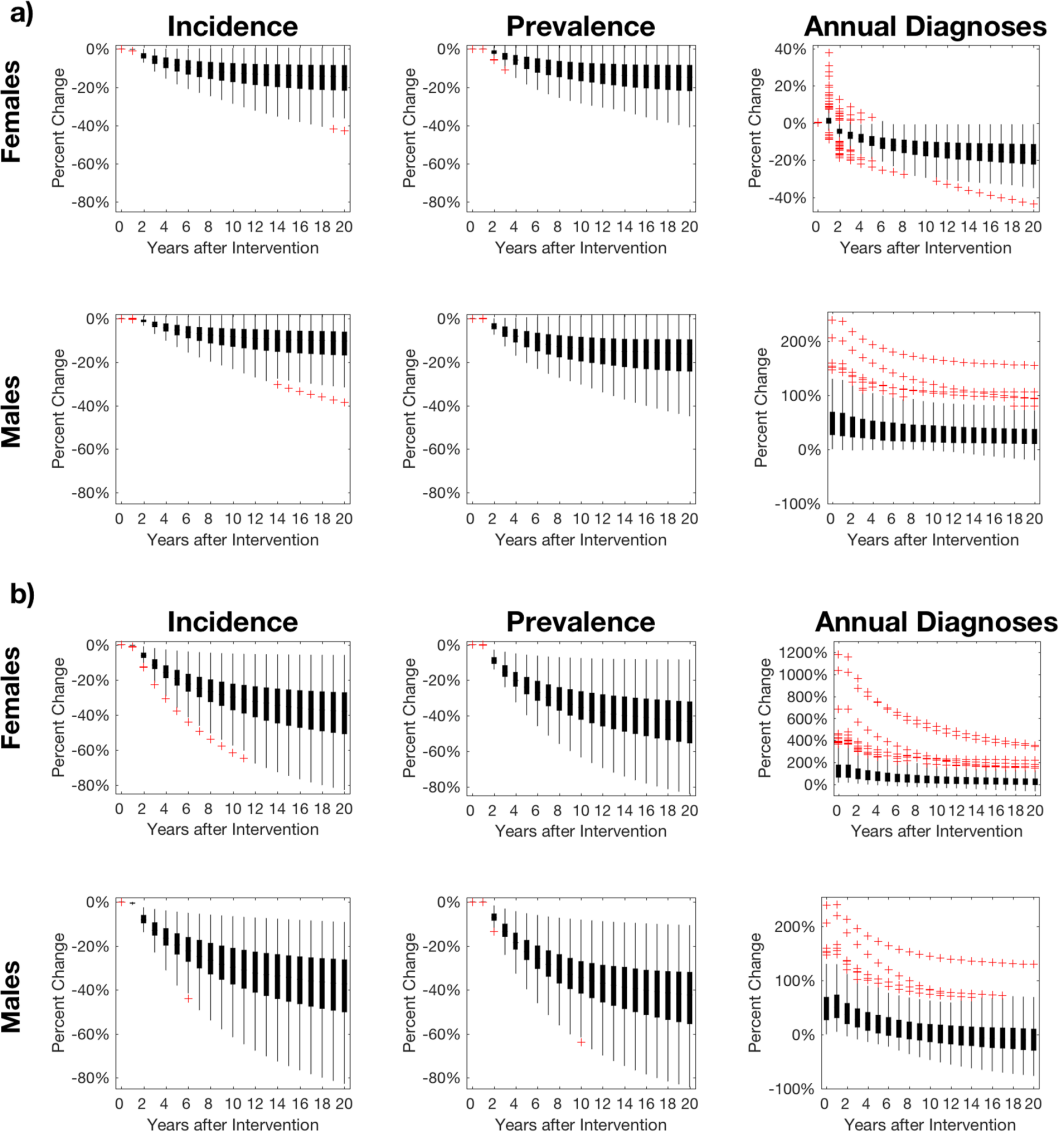
Effect of NAAT testing on incidence and prevalence of *M*. *genitalium* infection and rates of diagnosis. (**a**) NAAT testing of symptomatic men and symptomatic women in GP and GUM clinics, and of the 5% of asymptomatic men who were previously were screened in GUM with microscopy; (**b**) NAAT testing for all patients in GUM clinics plus symptomatic patients in GP clinics. Box plots show the mean, interquartile range, 95% range and outliers of the proportionate change in rates compared with baseline for each of the accepted parameter sets. Year 0 is the baseline. Note that in each scenario, (**a**) and (**b**), the vertical scales for changes in incidence are the same for both sexes but are different for changes in diagnoses. In both sexes, there is a reduction in incidence of infection, with incidence declining over time. The rate of diagnoses shows a different pattern from incidence: there is initially an increase, due to the increase in testing, followed by a decline, due to the consequent reduction in incidence and prevalence of infection. The patterns of changes in rates of diagnosis are different for each sex and differ between testing scenarios.

**Figure 5:**
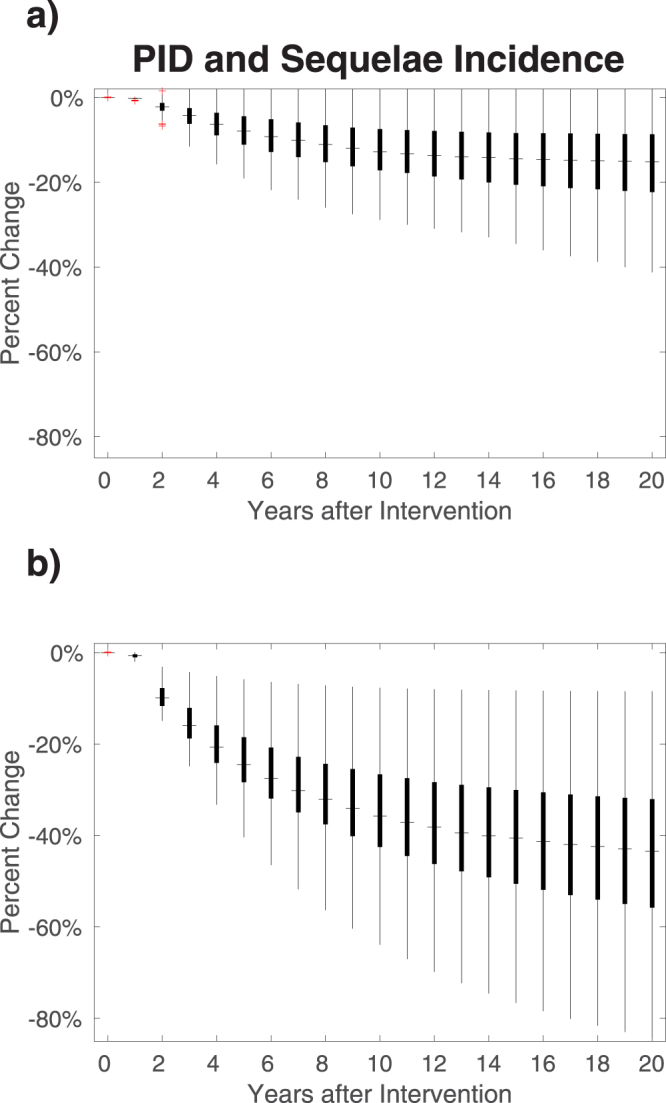
Impact on serious sequelae in women of introducing NAAT testing for *M*. *genitalium*. (**a**) NAAT testing symptomatic men and women in GP and GUM clinics, and NAAT testing of the 5% of asymptomatic men who were previously screened in GUM with microscopy; (**b**) NAAT testing of all patients in GUM clinics and symptomatic patients in GP clinics; Year 0 is the baseline. There is uncertainty in the magnitude of the effect due to uncertainty in natural history and behaviour parameter values, so results are presented as frequency distributions of proportionate changes in rates of PID due to *M*. *genitalium*. It takes at least several years for the full effect of the change to occur.

Introduction of NAAT testing leads to an increase in diagnosis and treatment, leading to a reduction in the incidence of infection, which declines over a sustained period, with uncertainty in natural history and behaviour parameters leading to uncertainty in the magnitude of the reduction (Figs 4 and 5). The impact on PID of NAAT testing is marked (Fig. 5, Table 4), particularly in the scenario where asymptomatic patients are screened because this identifies many more of the infections that occur compared with only testing symptomatic patients. NAAT testing enables detection of infection in women as well as men, detection of asymptomatic infection, and improved care of symptomatic patients because *M*. *genitalium* is treated specifically, rather than syndromic management being given for NCNGU/mucopurulent cervicitis.

Whereas introducing NAAT testing results in an immediate reduction in incidence which increases over time in both sexes, the effect on the rate of diagnoses is different. Initially there is an increase in diagnoses, due to the increase in testing, followed by a decline in diagnoses, due to the consequent reduction in prevalence and incidence of infection. The long-term effect on the diagnosis rate depends upon the testing scenario and differs for each sex. When NAAT testing is used for symptomatic patients (Figs 4a and 5a), the diagnosis rate in women increases by a relatively small amount and in the long-term falls below the baseline diagnosis rate prior to the intervention, whereas in men the initial increase in the diagnosis rate is proportionately larger, and in the long term, the diagnosis rate remains above baseline. When NAAT testing is used for symptomatic patients and asymptomatic patients in GUM (Figs 4b and 5b), the diagnosis rate in women increases by a relatively large amount and in the long-term remains slightly above the baseline diagnosis rate, whereas in men the initial increase in the diagnosis rate is proportionately smaller, and in the long term, the diagnosis rate remains slightly below baseline. The scenario presented in Figs (4b and 5b) is perhaps the one more likely to occur in practice, with the advent of multiplex NAAT tests meaning that asymptomatic patients tested for *C*. *trachomatis* and/or *N*. *gonorrhoeae* will often be tested automatically for *M*. *genitalium* as well.

Whilst in all cases NAAT testing reduced the incidence of *M*. *genitalium* infection and reduced the incidence of PID and other sequelae, it is important to note that uncertainty in *M*. *genitalium’s* natural history parameters leads to substantial uncertainty in the magnitude of these changes. In the case of the rate of diagnosis of *M*. *genitalium* infection there was uncertainty not only in the magnitude of the change but also in whether in the long term the diagnosis rate would be higher or lower (and it could be different for each sex). We calculated PRCCs for each parameter with respect to symptomatic male cases, epidemiologically treated male cases, and female prevalence, as well as for reductions in female and male incidence and PID prevalence (Tables 2, 3). The most influential parameters for each were rate of asymptomatic males seeking care, proportion of females who are symptomatic and female to male transmission probability. This analysis indicates that getting better estimates for these parameters will be key for maximally accurate assessment of the impact of NAAT testing roll-out.

## Discussion

We find that screening for *M*. *genitalium* using NAATs could lead to significant reductions in rates of PID in women due to *M*. *genitalium*, with cumulative incidence over 20 years reduced by 31.1% (95% range: 13.0–52.0%). This will reduce incidence of other serious sequelae arising from PID such as ectopic pregnancy and tubal factor infertility. Using NAAT testing for *M*. *genitalium* in general practice/community and specialist settings, instead of urethral smear microscopy testing of symptomatic men in GUM and syndromic management of both sexes, greatly reduces the incidence of infection and PID, particularly when symptomatic and asymptomatic patients are tested. This is for several reasons. Firstly, coverage of testing can be greater, as it can be offered to more patients (in community settings as well as GUM), and will likely be more acceptable to men than urethral smear microscopy. Secondly, women can be tested by NAAT, meaning that treatment of infection in women is not dependent upon either having symptomatic infection or being a notified partner of a man diagnosed with infection. Thirdly, detecting infection in women then enables contact tracing to find their male partners, further-reducing infection in the population. Moreover, with antibiotic resistance in *M*. *genitalium* causing increasing concern^2,27,54^, an important benefit of NAAT diagnosis of *M*. *genitalium* infection rather than syndromic management of NCNGU is that it allows use of a more-appropriate treatment regimen; NAATs can detect genetic determinants of drug resistance.

Importantly, our modelling shows that patterns observed in rates of diagnosis differ from patterns in underlying incidence, which needs to be taken into account when assessing surveillance data, as has previously been highlighted for gonorrhoea^55^: specifically, incidence falls immediately and remains lower than prior to intervention, whilst the diagnosis rate initially increases and then falls, and in the long-term the diagnosis rate may be lower or higher than prior to the intervention. A diagnosis rate that remains elevated may be interpreted wrongly as indicating a failure of the intervention or even a higher incidence of infection than prior to intervention.

To our knowledge, we are the first to use a transmission-dynamic model to synthesise evidence on the natural history and epidemiology of *M*. *genitalium*, and the impact of screening and treatment. We have identified key natural history parameters whose values are uncertain, which leads to considerable uncertainty in the magnitude of the effect of screening. Further research is required to obtain better estimates of these parameters before it can be determined how effective NAAT-based screening for *M*. *genitalium* is likely to be. Natural history studies of sexually-transmitted infections typically concentrate on women, due to concern about serious sequelae, but since most infected women become infected from men it is important to know parameter values for both sexes, to inform effective control policies. We note that results are not always as expected: in the case of *C*. *trachomatis*, established infections clear more slowly in men than women, which is the opposite of what is typically assumed^31^.

Our analysis shows that particularly important for *M*. *genitalium* transmission are the proportion of infections that are symptomatic (and proportion of those that are treated), duration of untreated infection, and infectivity, in both sexes. Measurement of these parameters will involve a variety of studies^56^, including analysis of surveillance data; it is possible to calculate population prevalence of infection based on rates of screening and diagnosis^57^. To maximise the value of surveillance data it is important to have a good understanding of the processes that lead to patients being tested and diagnosed to be able to make more-precise inferences^57^, e.g. what proportion of diagnosed infections are in symptomatic vs asymptomatic individuals, and what is each patient’s reason for testing, e.g. due to symptoms, being a notified partner of a diagnosed case, having been at perceived risk of infection and sought a test, or having been offered screening by a healthcare provider.

Combining data from contact tracing with whole-genome sequencing of isolates allows estimation of the timing of transmission from person to person, which provides lower-bound information on the duration of infection; this has been done for gonorrhoea and could be applied to *M*. *genitalium* ^58^. Estimates of the duration of untreated infection are best obtained from cohort studies in which individuals are followed over months or years without infections being treated, which have been performed for *M*. *genitalium* in women^11,59^ (see Smieszek & White 2016^60^ for a synthesis of those studies) but not men. Such studies are only possible where testing for and treating *M*. *genitalium* is not the standard of care. It is therefore vital to conduct studies urgently before screening and treating *M*. *genitalium* becomes common due to adoption of multiplex NAAT testing.

Multiplex NAATs that test for both *C*. *trachomatis* and *M*. *genitalium* provide an opportunity for large-scale surveillance, of *M*. *genitalium*, at minimal cost, through unlinked anonymous testing for *M*. *genitalium* of samples from patients who are screened for *C*. *trachomatis*, in order to identify risk groups in whom screening for *M*. *genitalium* would be justified. Additionally, diagnosis of NCNGU requires ruling-out of *C*. *trachomatis* and *N*. *gonorrhoeae*, so using a multiplex test that detects those organisms plus *M*. *genitalium* would be a simple way to monitor the proportion of NCNGU that is associated with *M*. *genitalium* and examine associations with age, sex, and location. Such studies are important since the positive predictive value (PPV) of the test depends upon the prevalence of infection in those screened, as well as test sensitivity and specificity, and only in groups where prevalence is sufficiently high is the PPV high enough to justify screening for treatment. It might be appropriate to target *M*. *genitalium* testing by age, or according to patient characteristics (e.g. those with symptoms, and/or with greater numbers of recent sexual partners)^11,61–63^. Geographic targeting may be appropriate, since it is likely that prevalence will vary geographically, as it does for *C*. *trachomatis* ^57^.

In conclusion, it is unclear at present whether screening for *M*. *genitalium* should be recommended, due to uncertainty in key natural history parameters identified in our analysis. The ongoing uncertainty in the impact of chlamydia screening highlights the need for caution. We hope that this work will enable empirical research activity to be focused where it will be most effective in informing public-health decision-making.

## Electronic supplementary material

Supplementary Information

## References

[1] McGowin CL, Anderson-Smits C (2011). Mycoplasma genitalium: An Emerging Cause of Sexually Transmitted Disease in Women. PLoS Pathogens.

[2] Jensen JS, Cusini M, Gomberg M, Moi H (2016). Background review for the 2016 European guideline on Mycoplasma genitalium infections. J Eur Acad Dermatology Venereol.

[3] Anagrius C, Loré B, Jensen JS (2005). Mycoplasma genitalium: prevalence, clinical significance, and transmission. Sex Transm Infect.

[4] Manhart LE (2003). Mucopurulent Cervicitis and Mycoplasma genitalium. J Infect Dis.

[5] Lis R, Rowhani-Rahbar A, Manhart LE (2015). *Mycoplasma genitalium* Infection and Female Reproductive Tract Disease: A Meta-analysis. Clin Infect Dis.

[6] Bjartling C, Osser S, Persson K (2012). Mycoplasma genitalium in cervicitis and pelvic inflammatory disease among women at a gynecologic outpatient service. Am J Obstet Gynecol.

[7] Cohen CR (2002). Association between Mycoplasma genitalium and acute endometritis. Lancet.

[8] Haggerty, C. L., Totten, P. A., Astete, S. G. & Ness, R. B. *Mycoplasma genitalium* among women with nongonococcal, nonchlamydial pelvic inflammatory disease. *Infect Dis Obstet Gynecol* **2006**, 10.1155/IDOG/2006/30184 (2006). 10.1155/IDOG/2006/30184 PMC1581464 17485798

[9] Haggerty CL (2008). Failure of cefoxitin and doxycycline to eradicate endometrial Mycoplasma genitalium and the consequence for clinical cure of pelvic inflammatory disease..

[10] Simms I, Hurtig A-K, Rogers PA, Hughes G, Fenton KA (2003). Surveillance of sexually transmitted infections in primary care. Sex Transm Infect.

[11] Oakeshott P (2010). Is Mycoplasma genitalium in Women the ‘New Chlamydia?’ A Community-Based Prospective Cohort Study. Clin Infect Dis.

[12] Clausen HF (2001). Serological investigation of Mycoplasma genitalium in infertile women. Hum Reprod.

[13] Svenstrup HF (2008). Mycoplasma genitalium, Chlamydia trachomatis, and tubal factor infertility-a prospective study. Fertil Steril.

[14] Walker J (2013). Mycoplasma genitalium incidence, organism load, and treatment failure in a cohort of young Australian women. Clin Infect Dis.

[15] Hillis SD (1993). Delayed care of pelvic inflammatory disease as a risk factor for impaired fertility. Am J Obstet Gynecol.

[16] Westrom L (1994). Sexually transmitted diseases and infertility. Sex Transm Dis.

[17] Westrom L (1995). Effect of pelvic inflammatory disease on fertility. Venereology.

[18] Bjartling C, Osser S, Persson K (2010). The association between Mycoplasma genitalium and pelvic inflammatory disease after termination of pregnancy. BJOG An Int J Obstet Gynaecol.

[19] Skov Jensen J (2004). Mycoplasma genitalium: The aetiological agent of urethritis and other sexually transmitted diseases. Journal of the European Academy of Dermatology and Venereology.

[20] Taylor-Robinson D, Horner PJ (2001). The role of Mycoplasma genitalium in non-gonococcal urethritis. Sex Transm Infect.

[21] Horner PJ, Taylor-Robinson D (2011). Association of Mycoplasma genitalium with balanoposthitis in men with non-gonococcal urethritis. Sex Transm Infect.

[22] Mavedzenge SN (2012). The association between Mycoplasma genitalium and HIV-1 acquisition in African women. AIDS.

[23] Ursi D, Crucitti T, Smet H, Ieven M (2016). Evaluation of the Bio-Rad Dx CT/NG/MG® assay for simultaneous detection of Chlamydia trachomatis, Neisseria gonorrhoeae and Mycoplasma genitalium in urine. Eur J Clin Microbiol Infect Dis.

[24] Manhart LE (2009). Has the Time Come to Systematically Test for Mycoplasma genitalium?. Sex Transm Dis.

[25] Manhart LE (2014). Diagnostic and Resistance Testing for Mycoplasma genitalium: What Will It Take?. Clin Infect Dis.

[26] Ross JDC, Jensen JS (2006). Mycoplasma genitalium as a sexually transmitted infection: implications for screening, testing, and treatment. Sex Transm Infect.

[27] Horner P, Blee K, Adams E (2014). Time to manage Mycoplasma genitalium as an STI: but not with azithromycin 1 g!. Curr Opin Infect Dis.

[28] World Health Organization. Global Health Sector Strategy on Sexually Transmitted Infections 2016–2021. Available at: http://www.who.int/reproductivehealth/publications/rtis/ghss-stis/en/. (Date of Access: 13/07/2017) (2016).

[29] Davies B, Anderson S-J, Turner KM, Ward H (2014). How robust are the natural history parameters used in chlamydia transmission dynamic models? A systematic review. Theor Biol Med Model.

[30] Price MJ (2016). The natural history of Chlamydia trachomatis infection in women: a multi-parameter evidence synthesis. Health Technol Assess.

[31] Lewis J, Price MJ, Horner PJ, White PJ (2017). Genital chlamydia trachomatis infections clear more slowly in men than women, but are less likely to become established. J Infect Dis.

[32] Hallett TB, White PJ, Garnett GP (2007). Appropriate evaluation of HIV prevention interventions: from experiment to full-scale implementation. Sex Transm Infect.

[33] Estcourt C (2016). The Ballseye programme: a mixed-methods programme of research in traditional sexual health and alternative community settings to improve the sexual health of men in the UK. Program Grants Appl Res.

[34] Garnett G, Mertz K, Finelli L (1999). The transmission dynamics of gonorrhoea: modelling the reported behaviour of infected patients from Newark, New Jersey. Philos Trans R Soc Lond B Biol Sci.

[35] Carne CA (2010). National audit of asymptomatic screening in UK genitourinary medicine clinics: clinic policies audit. Int J STD AIDS.

[36] Björnelius E (2008). Antibiotic treatment of symptomatic Mycoplasma genitalium infection in Scandinavia: a controlled clinical trial. Sex Transm Infect.

[37] Bradshaw CS (2006). Azithromycin Failure in Urethritis. Emerg Infect Dis.

[38] Jernberg E, Moghaddam A, Moi H (2008). Azithromycin and moxifloxacin for microbiological cure of Mycoplasma genitalium infection: an open study. Int J STD AIDS.

[39] Lau A (2015). The Efficacy of Azithromycin for the Treatment of Genital Mycoplasma genitalium: A Systematic Review and Meta-analysi. s. Clin Infect Dis.

[40] Adams EJ, Turner KME, Edmunds WJ (2007). The cost effectiveness of opportunistic chlamydia screening in England. Sex Transm Infect.

[41] Bradshaw, C. S. *et al*. Azithromycin failure in Mycoplasma genitalium urethritis. *Emerg Infect Dis* **12**, 1149–1152, https://doi.org/10.3201/eid1207.051558 (2006). 10.3201/eid1207.051558 PMC3291056 16836839

[42] Cassell JA, Brook MG, Mercer CH, Murphy S, Johnson AM (2003). Treating sexually transmitted infections in primary care: a missed opportunity?. Sex Transm Infect.

[43] Cassell J (2003). Partner notification in primary care. Sex Transm Infect.

[44] Jensen JS, Björnelius E, Dohn B, Lidbrink P (2004). Comparison of First Void Urine and Urogenital Swab Specimens for Detection of Mycoplasma genitalium and Chlamydia trachomatis by Polymerase Chain Reaction in Patients Attending a Sexually Transmitted Disease Clinic. Sex Transm Dis.

[45] Mercer CH (2009). Who has sex with whom? Characteristics of heterosexual partnerships reported in a national probability survey and implications for STI risk. Int J Epidemiol.

[46] Mercer CH (2007). How much do delayed healthcare seeking, delayed care provision, and diversion from primary care contribute to the transmission of STIs?. Sex Transm Infect.

[47] Irwin DE (1999). Self-reported sexual activity and condom use among symptomatic clients attending STD clinics. Sex Transm Dis.

[48] Fortenberry JD (1997). Health care seeking behaviors related to sexually transmitted diseases among adolescents. Am J Public Heal.

[49] Bradshaw CS, Chen MY, Fairley CK (2008). Persistence of Mycoplasma genitalium Following Azithromycin Therapy. PLoS One.

[50] Health Protection Agency. KC60 GUM Clinic Surveillance. Available at: http://webarchive.nationalarchives.gov.uk/20090709063949/http://www.hpa.org.uk/web/HPAweb&Page&HPAwebAutoListName/Page/1201094610372. (Date of Access: 17/07/2017) (2009).

[51] Ishihara S, Yasuda M, Ito SI, Maeda SI, Deguchi T (2004). Mycoplasma genitalium urethritis in men. in. International Journal of Antimicrobial Agents.

[52] Mena L, Wang X, Mroczkowski TF, Martin DH (2002). Mycoplasma genitalium Infections in Asymptomatic Men and Men with Urethritis Attending a Sexually Transmitted Diseases Clinic in New Orleans. Clin Infect Dis.

[53] Blower SM, Dowlatabadi H (1994). Sensitivity and Uncertainty Analysis of Complex Models of Disease Transmission: An HIV Model, as an Example. Int Stat Rev Int Stat.

[54] Jensen JS, Cusini M, Gomberg M, Moi H (2016). 2016 European guideline on *Mycoplasma genitalium* infections. J Eur Acad Dermatology Venereol.

[55] White PJ, Ward H, Cassell JA, Mercer CH, Garnett GP (2005). Vicious and Virtuous Circles in the Dynamics of Infectious Disease and the Provision of Health Care: Gonorrhea in Britain as an Example. J Infect Dis.

[56] Grad YH, Goldstein E, Lipsitch M, White PJ (2016). Improving Control of Antibiotic-Resistant Gonorrhea by Integrating Research Agendas Across Disciplines: Key Questions Arising From Mathematical Modeling. J Infect Dis.

[57] Lewis J, White PJ (2017). Estimating Local Chlamydia Incidence and Prevalence Using Surveillance Data. Epidemiology.

[58] Didelot X (2016). Genomic Analysis and Comparison of Two Gonorrhea Outbreaks. MBio.

[59] Vandepitte J (2013). Natural history of Mycoplasma genitalium Infection in a Cohort of Female Sex Workers in Kampala, Uganda. Sex Transm Dis.

[60] Smieszek T, White PJ (2016). Apparently-different clearance rates from cohort studies of Mycoplasma genitalium are consistent after accounting for incidence of infection, recurrent infection, and study design. PLoS One.

[61] Andersen B (2007). Mycoplasma genitalium: prevalence and behavioural risk factors in the general population. Sex Transm Infect.

[62] Cohen CR (2007). Mycoplasma genitalium Infection and Persistence in a Cohort of Female Sex Workers in Nairobi, Kenya. Sex Transm Dis.

[63] Svenstrup HF (2014). A cross-sectional study of Mycoplasma genitalium infection and correlates in women undergoing population-based screening or clinic-based testing for Chlamydia infection in London. BMJ Open.

[64] Johnson AM (2001). Sexual behaviour in Britain: partnerships, practices, and HIV risk behaviours. Lancet.

[65] Mercer CH (2013). Changes in sexual attitudes and lifestyles in Britain through the life course and over time: findings from the National Surveys of Sexual Attitudes and Lifestyles (Natsal). Lancet (London, England).

[66] Keane FEA, Thomas BJ, Gilroy CB, Renton A, Taylor-Robinson D (2000). The association of Chlamydia trachomatis and Mycoplasma genitalium with non-gonococcal urethritis: observations on heterosexual men and their female partners. Int J STD AIDS.

[67] Quinn TC (1996). Epidemiologic and Microbiologic Correlates of Chlamydia trachomatis Infection in Sexual Partnerships. JAMA.

[68] Thurman AR (2010). Mycoplasma genitalium symptoms, concordance and treatment in high-risk sexual dyads. Int J STD AIDS.

[69] Falk L, Fredlund H, Jensen JS (2004). Symptomatic urethritis is more prevalent in men infected with Mycoplasma genitalium than with Chlamydia trachomatis. Sex Transm Infect.

[70] Lewis, D. A. *et al*. The burden of asymptomatic sexually transmitted infections among men in Carletonville, South Africa: implications for syndromic management. *Sex Transm Infect* **84**, 371–376, 10.1136/sti.2008.029751 (2008). 10.1136/sti.2008.029751 18385225

[71] Saunders JM (2012). Where do young men want to access STI screening? A stratified random probability sample survey of young men in Great Britain. Sex Transm Infect.

[72] Cassell JA (2006). Trends in sexually transmitted infections in general practice 1990-2000: population based study using data from the UK general practice research database. BMJ.

[73] Smith R (2003). Poor sensitivity and consistency of microscopy in the diagnosis of low grade non-gonococcal urethritis. Sex Transm Infect.

[74] Stellrecht KA, Woron AM, Mishrik NG, Venezia RA (2004). Comparison of multiplex PCR assay with culture for detection of genital mycoplasmas. J Clin Microbiol.

[75] McClean, H., Radcliffe, K., Sullivan, A. & Ahmed-Jushuf, I. 2012 BASHH statement on partner notification for sexually transmissible infections. *Int J STD AIDS* **24**, 253–61, doi:10.1177/0956462412472804 (2013). 10.1177/0956462412472804 23970656

[76] Turner KME (2006). Modelling the effectiveness of chlamydia screening in England. Sex Transm Infect.

