# Supplementary material for: Should we screen for the sexually-transmitted infection Mycoplasma genitalium? Evidence synthesis using a transmission-dynamic model
Source: Sci Rep. 2017 Nov 23;7:16162. doi: 10.1038/s41598-017-16302-8 (PMC5700964; doi:10.1038/s41598-017-16302-8)

Should we screen for the sexually-transmitted infection *Mycoplasma genitalium*? Evidence synthesis using a transmission-dynamic model

Ruthie Birger, John Saunders, Claudia Estcourt, Andrew John Sutton, Catherine H Mercer, Tracy Roberts, Peter J White

Model Equations

$$\begin{aligned}
\frac{dS(i,j)}{dt} &= \mu N(i,j) - \lambda(i,j)S(i,j) + \gamma(i)I_A(i,j) + p_{GUM}(i) \times sens_{M/P} \times (1-\zeta(i)) \times \eta\sigma(i)I_A(i,j) + \\
&\quad \frac{p_{GUM}(i) \times sens_{M/P} \times (1-\zeta(i)) \times I_T(i,j)}{d_{inf}(i)} + \frac{p_{GUM}(i) \times (1-sens_{M/P}) \times (1-\zeta(i)) \times I_T(i,j)}{d_{inf}(i)} + \\
&\quad \frac{p_{GP}(i) \times (1-\zeta(i)) \times I_T(i,j)}{d_{inf}(i)} + \gamma(i) \times I_X(i,j) + \gamma_F \times I_F(i,j) + \frac{I_F(i,j)}{d_{inf2}} + \\
&\quad \gamma_{PID}(i,j) \times (P_A(i,j) + P(i,j)) + \frac{P_C(i,j)}{d_{tp}} + (1-\zeta(i)) \times (PN_A(i,j) + PN_X(i,j) + PN_T(i,j)) - \mu S(i,j) \\
\frac{dE(i,j)}{dt} &= \lambda(i,j) \times S(i,j) - \frac{E(i,j)}{d_{lat}} - \mu E(i,j) \\
\frac{dI_A(i,j)}{dt} &= (1-z(i)) \times \frac{E(i,j)}{d_{lat}} - \gamma(i) \times I_A(i,j) - p_{GUM}(i) \times sens_{M/P} \times \eta\sigma(i)I_A(i,j) + \\
&\quad p_{GUM}(i) \times sens_{M/P} \times \zeta(i)\eta\sigma(i)I_A(i,j) - PN_A(i,j) + \zeta(i) \times PN_A(i,j) - \\
&\quad \psi_p(i,j) \times I_A(i,j) - \mu I_A(i,j) \\
\frac{dI_X(i,j)}{dt} &= z(i) \times (1-\rho(i)) \times \frac{E(i,j)}{d_{lat}} - \gamma(i)I_X(i,j) - PN_X(i,j) - \psi_p(i,j)I_X(i,j) - \mu I_X(i,j) \\
\frac{dI_T(i,j)}{dt} &= z(i) \times \rho(i) \times \frac{E(i,j)}{d_{lat}} - PN_T(i,j) - \frac{I_T(i,j)}{d_{inf}(i)} - \mu I_T(i,j) \\
\frac{dI_F(i,j)}{dt} &= \frac{p_{GUM}(i) \times sens_{M/P} \times \zeta(i) \times I_T(i,j)}{d_{inf}(i)} + \frac{p_{GUM}(i) \times (1-sens_{M/P}) \times \zeta(i) \times I_T(i,j)}{d_{inf}(i)} + \\
&\quad \frac{p_{GP}(i) \times \zeta(i) \times I_T(i,j)}{d_{inf}(i)} - \psi_p(i,j)I_F(i,j) - \gamma_F I_F(i,j) - \\
&\quad \frac{I_F(i,j)}{d_{inf2}} + \zeta(i) \times (PN_X(i,j) + PN_T(i,j)) - \mu I_F(i,j) \\
\frac{dP_A(i,j)}{dt} &= (1-y) \times \psi_p(i,j) \times (I_A(i,j) + I_X(i,j) + I_F(i,j)) - \gamma_{PID}(i,j) \times P_A(i,j) - \mu P_A(i,j) \\
\frac{dP(i,j)}{dt} &= y \times \psi_p(i,j) \times (I_A(i,j) + I_X(i,j) + I_F(i,j)) - \gamma_{PID}(i,j) \times P(i,j) - \frac{P(i,j)}{d_{PT}} - \mu P(i,j) \\
\frac{dP_C(i,j)}{dt} &= \frac{P(i,j)}{d_{PT}} - \frac{P_C(i,j)}{d_{tp}} - \mu P_C(i,j)
\end{aligned}$$

Where *S* is susceptible; *E*, latent; I_A, infected asymptomatic; I_X, infected symptomatic (not seeking treatment); I_T, infected symptomatic (seeking treatment); I_F, treatment failures; P_A, PID not seeking treatment; *P*, PID seeking treatment; P_C, PID in treatment. PN_h terms represent the number of Partner Notified individuals; λ, force of infection; μ, rate of aging out of sexual activity; γ, natural recovery rates; *η*, proportion screened when attending GUM clinics; σ, rate of care-seeking; p_{GUM}, the proportion of those who seek treatment, who seek it through GUM; p_{GP}, the proportion of those who seek treatment, who seek it through general practice (derived below); *ρ*, proportion symptomatic who seek treatment; $sens_{M/P}$, sensitivity of microscopy test – men only (M), or NAAT (P); ψ_P, proportion of women progressing to PID; *z*, proportion of infections symptomatic; *y*, proportion of PID cases symptomatic; ς, treatment failure rate; and *d* terms, duration of disease stages ($d_{inf} = d_{seek} + d_{care}$). *i* and *j* indicate sex and activity class, respectively.

p_{GUM} and p_{GP} are composite calculations from the proportions of individuals who seek care who go straight to GUM and those who go to GUM via GP:

$p_{GUM} = p_{GUMD} + p_{GPGUM} \times (1 - p_{GUMD})$

$p_{GP} = (1 - p_{GPGUM}) \times (1 - p_{GUMD})$

The force of infection, *λ(i,j)*, is calculated through several steps.

$\lambda(i,j) = r(i,j)\Sigma_k\, \Omega(i,j,k)$

where $\Omega(i,j,k)$ is a term combined of a prevalence estimate, a mixing matrix coefficient, and a per-partnership transmission probability:

$$\Omega(i,j,k) = \beta(i,j,k) \times p(i,j,k) \times \frac{I_A(i',k) + I_X(i',k) + (1-\alpha) \times (I_T(i',k) + I_F(i',k)) + P(i',k)}{N(i',k)}$$

The mixing matrix is calculated as follows:

$$p(i,j,k) = \epsilon\delta_{ij} + (1-\epsilon)\frac{r(i',k)N(i',k)}{\sum_s r(i',s)N(i',s)}$$

The per-partnership transmission probability is composed of per-sex-act transmission probability, number of sex acts, and partner change rate:

$$\beta(i,j,k) = 1 - \left(1 - \phi(i)\right)\frac{q}{\max[r(i,j), r(i',k), \gamma(i')]}$$

where

α is reduction in sexual activity when care-seeking

ε is the assortativeness coefficient, varied continuously in the range (0,1), where 0 = completely random, 1 = completely assortative.

δ_{ij} is the Kronecker δ: $\delta_{ij} = 1$ when $i=j$, 0 otherwise.

$r(i,j)$ is the annual partner change rate for sex i, class j

$\phi(i)$ is per-sex-act transmission probability (differing for m-to-f and f-to-m)

q is the sex-act frequency (same for all activity classes)

Partner Notification

$$PN_{gum_h}(i,j) = \sum_k f_{GUM} p_{GUM} \left[C_h(i,j,k)\left(\sigma(i)\eta I_A(i',k) + \frac{1}{d_{inf}} I_T(i',k) + \frac{1}{d_{inf_{PID}}} P(i',k)\right) + \sigma(i)\eta(1-\nu)\left(S(i',k)L_{S_h} + E(i',k)L_{E_h}\right)\right]$$

$$PN_{gp_h}(i,j) = \sum_k f_{GP} p_{GP} C_h(i,j,k)\left(\frac{1}{d_{inf}} I_T(i',k) + \frac{1}{d_{inf_{PID}}} P(i',k)\right)$$

$$C_h(i,j,k) = L_{I_h}(i,j,k) + M_h(i,j,k)$$

Where h indicates category of infections (i.e. Asymptomatic, Symptomatic, or Symptomatic Treated);

i indicates sex and i' opposite sex; j,k are activity classes;

f_{GUM}, f_{GP} are the rates of contact tracing in GUM and GP settings, respectively;

$C_h(i,j,k)$ is total partners traced (i.e. partners of sex i, class j traced from index of sex i', class k),

which is the sum of

L_h, partners infected before index case (one of whom infected the index case) and

M_h, partners infected by the index case.

L_{E_h}, L_{S_h} are infected partners traced from false positives diagnosed in GUM,

while

L_{I_h} are infected partners traced from true positives diagnosed in GUM or GP.

$$L_{I_A}(i,j,k) = r(i,j)T_M p(i,j,k)\frac{1}{r(i,j)}\left[\exp\left(-lag_2(p_{GUM}\eta\sigma(i) + \gamma(i) + \psi_{P(i,j)})\right)\right.$$
$$\left. + \exp(-lag_1(p_{GUM}\eta\sigma(i) + \gamma(i) + \psi_P(i,j)))\right] \times \max\left[(r(i,j) - 1), 1\right] \times \frac{I_A(i',k)}{N(i',k)}$$

$$L_{I_X}(i,j,k) = r(i,j)T_M p(i,j,k)\frac{1}{r(i,j)}\left[\exp\left(-lag_2(\gamma(i) + \psi_{P(i,j)}\right)\right.$$
$$\left. + \exp\left(-lag_1(\gamma(i) + \psi_P(i,j))\right)\right] \times \max\left[(r(i,j) - 1), 1\right] \times \frac{I_A(i',k)}{N(i',k)}$$

$$L_{I_T}(i,j,k) = r(i,j)T_M p(i,j,k)\frac{1}{r(i,j)}\left[\exp\left(-lag_2\left(\frac{1}{d_{inf}} + \zeta\right)\right)\right.$$
$$\left. + \exp\left(-lag_1\left(\frac{1}{d_{inf}} + \zeta\right)\right)\right] \times \max\left[(r(i,j) - 1), 1\right] \times \frac{I_T(i',k)}{N(i',k)}$$

$$L_{E_h}(i,j,k) = r(i,j)d_{lat} p(i,j,k)\frac{1}{r(i,j)}\left[\exp\left(-lag_2(p_{GUM}\eta\sigma(i) + \gamma(i) + \psi_{P(i,j)})\right)\right.$$
$$\left. + \exp\left(-lag_1 p_{GUM}\eta\sigma(i) + \gamma(i) + \psi_P(i,j)\right)\right] \times \max\left[(r(i,j) - 1), 1\right] \times \frac{I_h(i',k)}{N(i',k)}$$

$$L_{E_X}(i,j,k) = r(i,j)d_{lat} p(i,j,k)\frac{1}{r(i,j)}\left[\exp\left(-lag_2(\gamma(i) + \psi_{P(i,j)}\right)\right.$$
$$\left. + \exp\left(-lag_1(\gamma(i) + \psi_P(i,j))\right)\right] \times \max\left[(r(i,j) - 1), 1\right] \times \frac{I_A(i',k)}{N(i',k)}$$

$$L_{E_T}(i,j,k) = r(i,j)d_{lat} p(i,j,k)\frac{1}{r(i,j)}\left[\exp\left(-lag_2\left(\frac{1}{d_{inf}} + \zeta\right)\right)\right.$$
$$\left. + \exp\left(-lag_1\left(\frac{1}{d_{inf}} + \zeta\right)\right)\right] \times \max\left[(r(i,j) - 1), 1\right] \times \frac{I_T(i',k)}{N(i',k)}$$

$$L_{S_A}(i,j,k) = r(i,j)T_M p(i,j,k)\frac{1}{r(i,j)}[\exp\big(-lag_2(p_{GUM}\eta\sigma(i) + \gamma(i) + \psi_{P(i,j)})\big)$$
$$+ \exp(-lag_1(p_{GUM}\eta\sigma(i) + \gamma(i) + \psi_P(i,j)))] \times \frac{I_A(i',k)}{N(i',k)}$$

$$L_{S_X}(i,j,k) = r(i,j)T_M p(i,j,k)\frac{1}{r(i,j)}[\exp\big(-lag_2(\gamma(i) + \psi_{P(i,j)}\big)$$
$$+ \exp\big(-lag_1(\gamma(i) + \psi_P(i,j))\big)] \times \frac{I_A(i',k)}{N(i',k)}$$

$$L_{S_T}(i,j,k) = r(i,j)T_M p(i,j,k)\frac{1}{r(i,j)}[\exp\,(-lag_2\left(\frac{1}{d_{inf}} + \zeta\right))$$
$$+ \exp\left(-lag_1\left(\frac{1}{d_{inf}} + \zeta\right)\right)] \times \frac{I_T(i',k)}{N(i',k)}$$

$$M_A(i,j,k) = r(i,j)\big(1 - z(i)\big)T_A \exp(-lag_3(p_{GUM}\eta\sigma(i) + \gamma(i)$$
$$+ \psi_P(i,j)))\ \beta(i,j,k)p(i,j,k)\frac{S(i',k)}{N(i',k)}$$

$$M_X(i,j,k) = r(i,j)\big(1 - \rho\ (i)\big)z(i)T_X \exp(-lag_3(\gamma(i) + \psi_P(i,j)))\ \beta(i,j,k)p(i,j,k)\frac{S(i',k)}{N(i',k)}$$

$$M_T(i,j,k) = r(i,j)\rho(i)z(i)\ T_T \exp(-lag_3\left(\frac{1}{d_{inf}} + \zeta\right))\ \beta(i,j,k)p(i,j,k)\frac{S(i',k)}{N(i',k)}$$

$$T_h = \min[d_{look}, dur_h]$$

$$dur_A = \frac{1}{\gamma(i) + \psi_{P(i)} + p_{GUM}\eta\sigma(i)}$$
$$dur_X = \frac{1}{\gamma(i) + \psi_P(i)}$$
$$dur_T = \frac{1}{\frac{1}{d_{inf}} + \zeta}$$

Where lag$_1$= $\tau + d_{look}$, mean delay in treatment of contacts + look-back period

lag$_2$= $\tau + d_{lat} + d_{seek} + d_{care}$, mean delay in treatment of contacts + latency period + time to seeking care + time receiving care

lag$_3$= $\tau - d_{lat} + d_{seek} + d_{care}$, mean delay in treatment of contacts - latency period + time to seeking care + time receiving care

and d_{look} is the look-back period for partner notification (60 days);

dur_h is average duration of infection in infection category *h*;

β is transmission probability;

lags are the various lags associated with partner notification,

comprising

τ , time to contact partners and

$d_{lat}, d_{seek}, d_{care}$, duration of latent, time to seeking care, and time to getting care respectively.

Likelihood Function

The model was calibrated to data on Male symptomatic diagnoses and epidemiologically treated cases (asymptomatic diagnoses), and female prevalence. As described in the main text, Latin Hypercube sampling was used to generate parameter sets, which were used to produce model runs. Fitting weights of parameter sets were determined by the following likelihood functions. For the male symptomatic and asymptomatic diagnoses, a Poisson likelihood was used:

$LLK(\theta) = -\theta + \log(\theta)\, Y$

Note: Y! was removed from the equation as it is constant across calculations, and Y is large enough to make the factorial unwieldy.

For female prevalence, a binomial likelihood was used:

$LLK(\theta) = \theta^* (Y^*\log(\theta)) + (1-Y)^*\log(1-\theta)$

Supplementary Tables

In scenario analysis, we varied rate that women with *M. genitalium* infections progress to PID (ψ), using low, medium, and high estimates of PID progression rate (0.022, 0.044, 0.09 respectively), corresponding to proportions of untreated infections progressing to PID of 2.1%, 4.5%, and 8.5%. The percentage change in cumulative incidence was essentially the same across the scenarios, at 31.2% (95% range:13.1%-51.8%) for $\psi = 0.022$, and 30.1% (95% range: 12.6%-52.1) for $\psi = 0.090$. Tables S1, S2 correspond to Table 4 in the main text and show the results for lower and higher progression rates.

Table S1 Numbers of cases of serious sequelae in women due to *M. genitalium* in the different scenarios, over 20 years, for 2.5% of infections progressing to PID ($\psi = 0.022$). Figures are averages of results obtained using the accepted parameter sets, weighted by the likelihood of each parameter set. The Difference columns report the difference between the baseline and intervention scenarios, calculated using the likelihood-weighted mean estimates, and the 95% ranges of the differences.

**Cumulative incidence**	**Mean**	**Weighted mean**	**Median**	**95% range**	**Difference**	
					**Mean**	**95% range**
Pelvic Inflammatory Disease						
Baseline	157,000	156,000	150,000	69,000–273,000	-	-
NAAT testing for symptomatic patients	140,000	138,000	132,000	64,200–258,000	17,100	~0–44,700
NAAT testing for all patients	111,000	109,000	107,000	38,300–226,000	46,400	20,800–77,800
Tubal Factor Infertility						
Baseline	9,400	9,300	9,000	4,100–16,400	-	-
NAAT testing for symptomatic patients	8,400	8,300	7,900	3,800–15,500	1,000	~0–2,700
NAAT testing for all patients	6,600	6,500	6,200	2,300–13,600	2,800	1,200–4,700
Ectopic Pregnancy						
Baseline	6,300	6,200	6,000	2,800–10,900	-	-
NAAT testing for symptomatic patients	5,600	5,500	5,300	2,600–10,300	700	~0–1,800
NAAT testing for all patients	4,400	4,400	4,100	1,500–9,100	1,900	800–3,100

Table S2 Numbers of cases of serious sequelae in women due to *M. genitalium* in the different scenarios, over 20 years, for 8.5% of infections progressing to PID ($\psi = 0.090$). Figures are averages of results obtained using the accepted parameter sets, weighted by the likelihood of each parameter set. The Difference columns report the difference between the baseline and intervention scenarios, calculated using the likelihood-weighted mean estimates, and the 95% ranges of the differences.

**Cumulative incidence**	**Mean**	**Weighted mean**	**Median**	**95% range**	**Difference**	
					**Mean**	**95% range**
Pelvic Inflammatory Disease						
Baseline	610,000	605,000	571,000	256,000–1,120,000	-	-
NAAT testing for symptomatic patients	543,000	538,000	505,000	236,000–1,040,000	66,900	~0–173,00
NAAT testing for all patients	439,000	433,000	395,000	152,000–935,000	171,000	76,100–287,000
Tubal Factor Infertility						
Baseline	36,600	36,300	34,300	15,400–67,400	-	-
NAAT testing for symptomatic patients	32,600	32,300	30,300	14,200–62,500	5,000	~0–10,400
NAAT testing for all patients	26,300	26,000	23,700	9,100–56,100	10,200	4,600–17,200
Ectopic Pregnancy						
Baseline	24,400	24,200	22,800	10,200–44,900	-	-
NAAT testing for symptomatic patients	21,700	21,500	20,200	9,400–41,700	2,700	~0–6,900
NAAT testing for all patients	17,600	17,300	15,800	6,100–37,400	6,800	3,000–11,500

Supplementary Figures

Trellis Plots

Figure S1a-d: These figures show a trellis plot of the parameter values. This large plot occupies 4 pages and is divided into quadrants which are arranged as follows: (a) top left, (b) top right, (c) bottom left, (d) bottom right. The diagonals in Figures S1a and d show histograms for the fitted values of each parameter (comparable to the violin plots in Figure 2 in the main text), while the off-diagonals show scatter plots of the fitted values for each pairwise combination of parameters. There is little correlation between parameter values, with the exception of $\rho(f)$ and f_{gum}.

a)

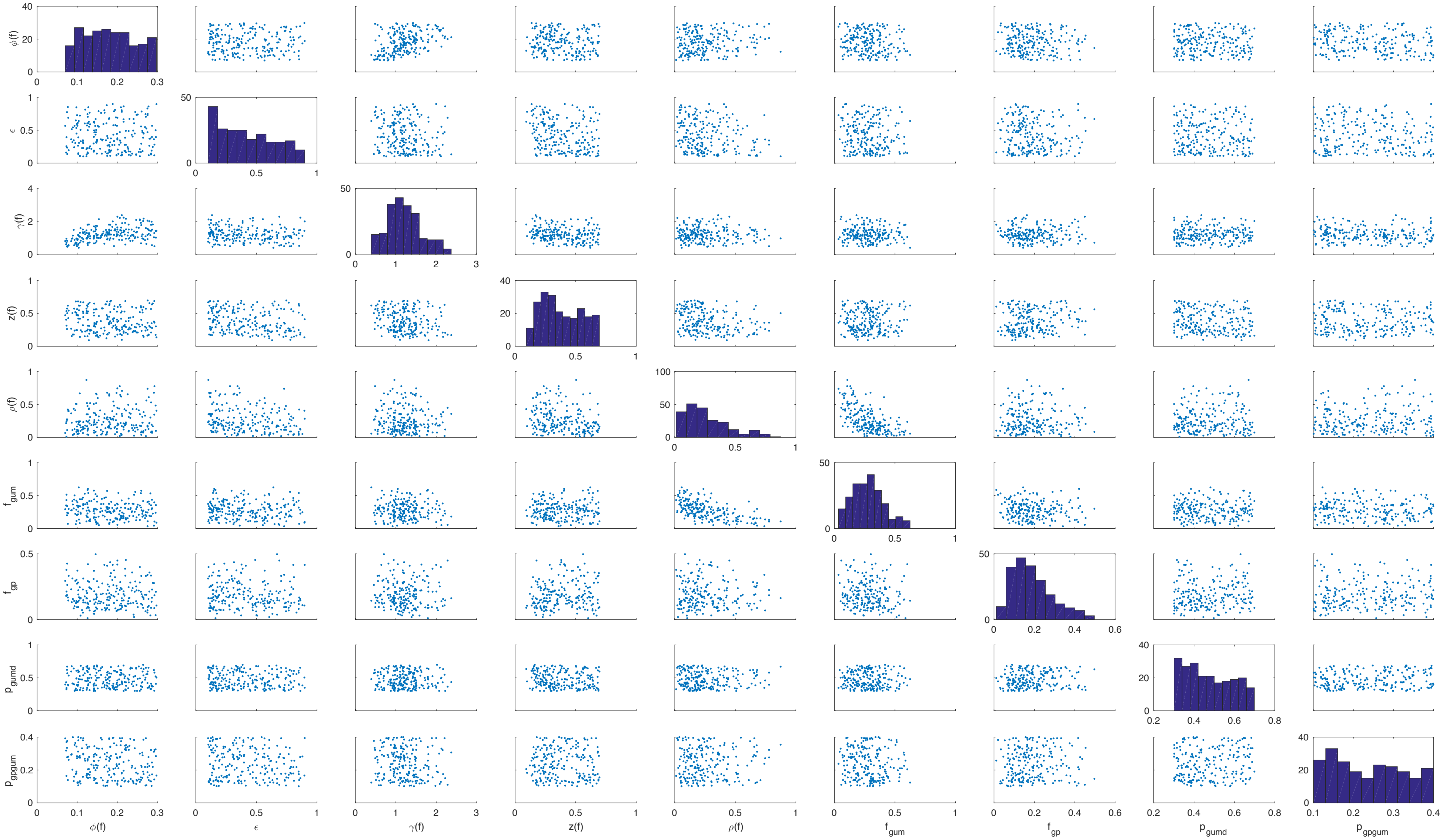

b)

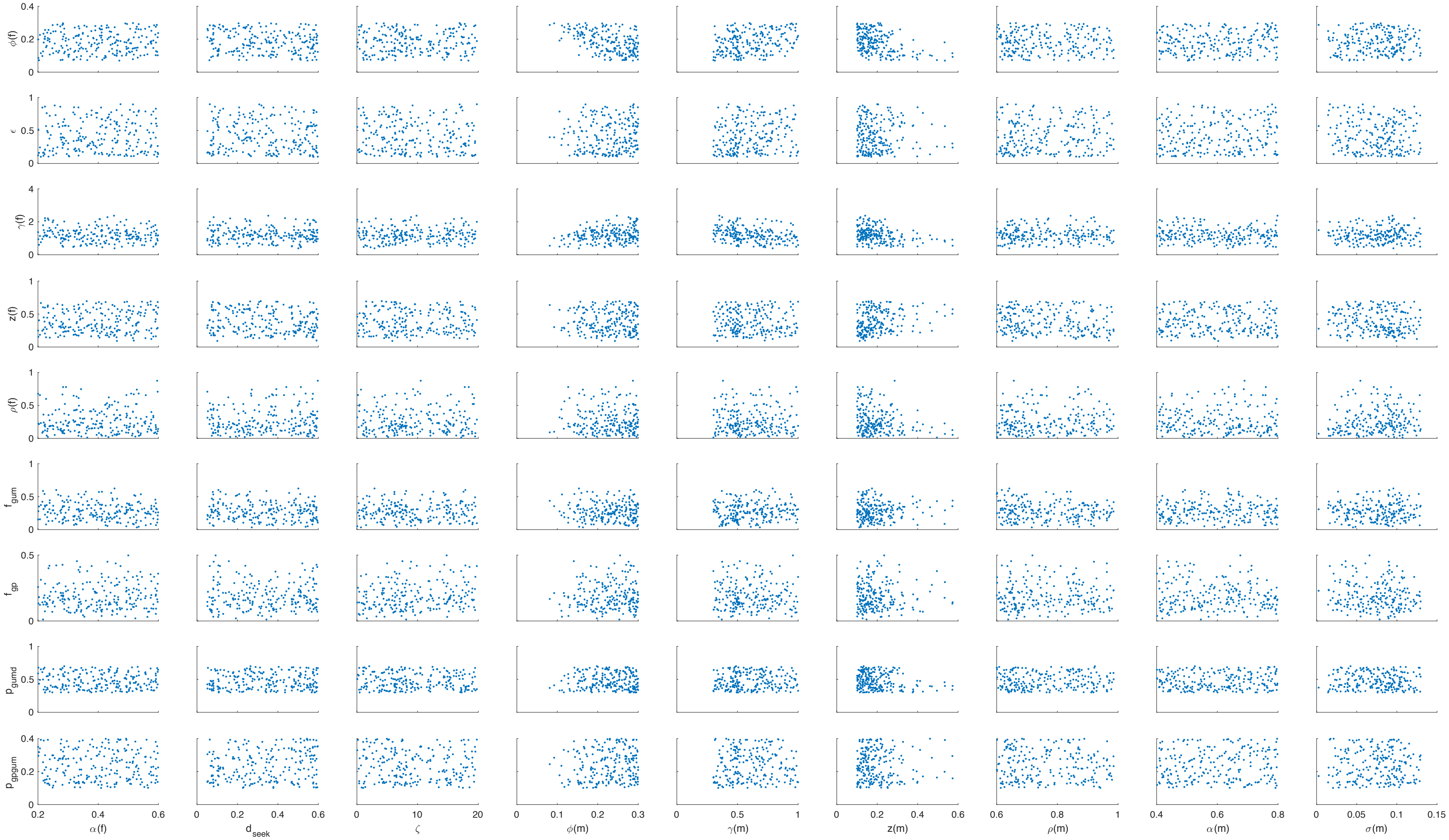

c)

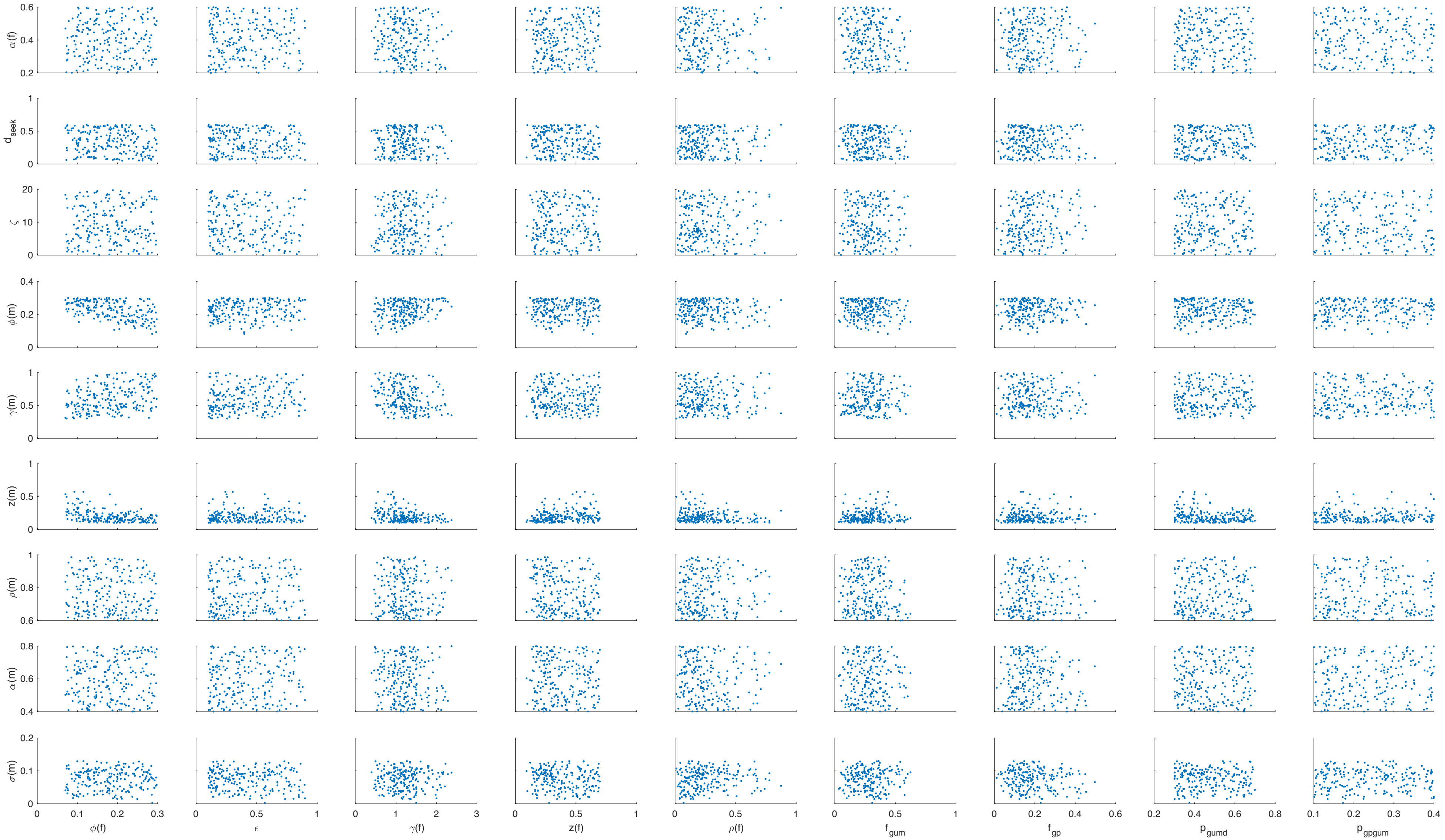

d)

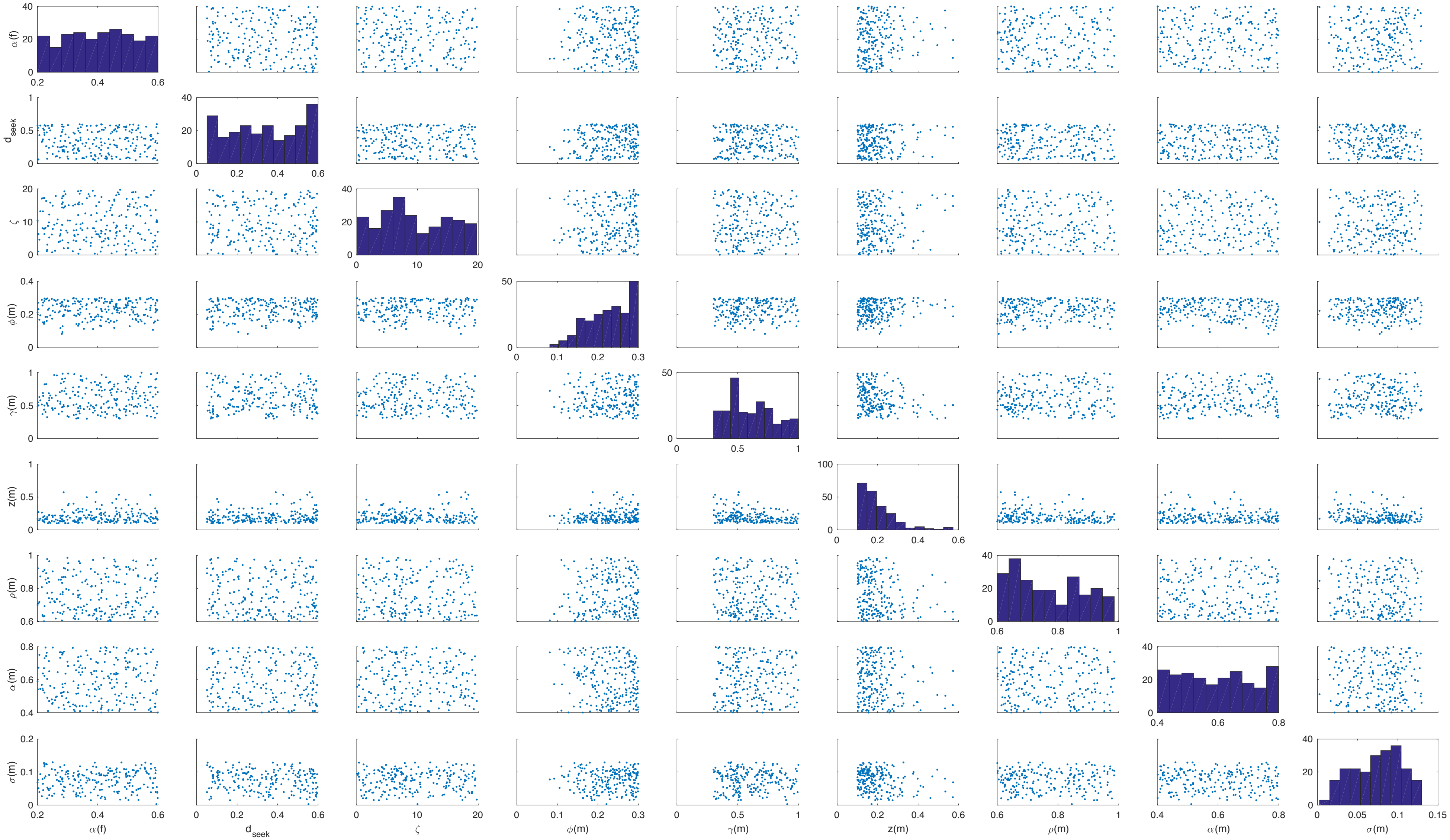

Figure S2: Trellis plots of equilibrium values for prevalence, incidence and annual diagnoses in each sex. All combinations are roughly positively correlated, as expected, particularly male prevalence, incidence, and diagnoses, and incidence in one sex and prevalence in the other.

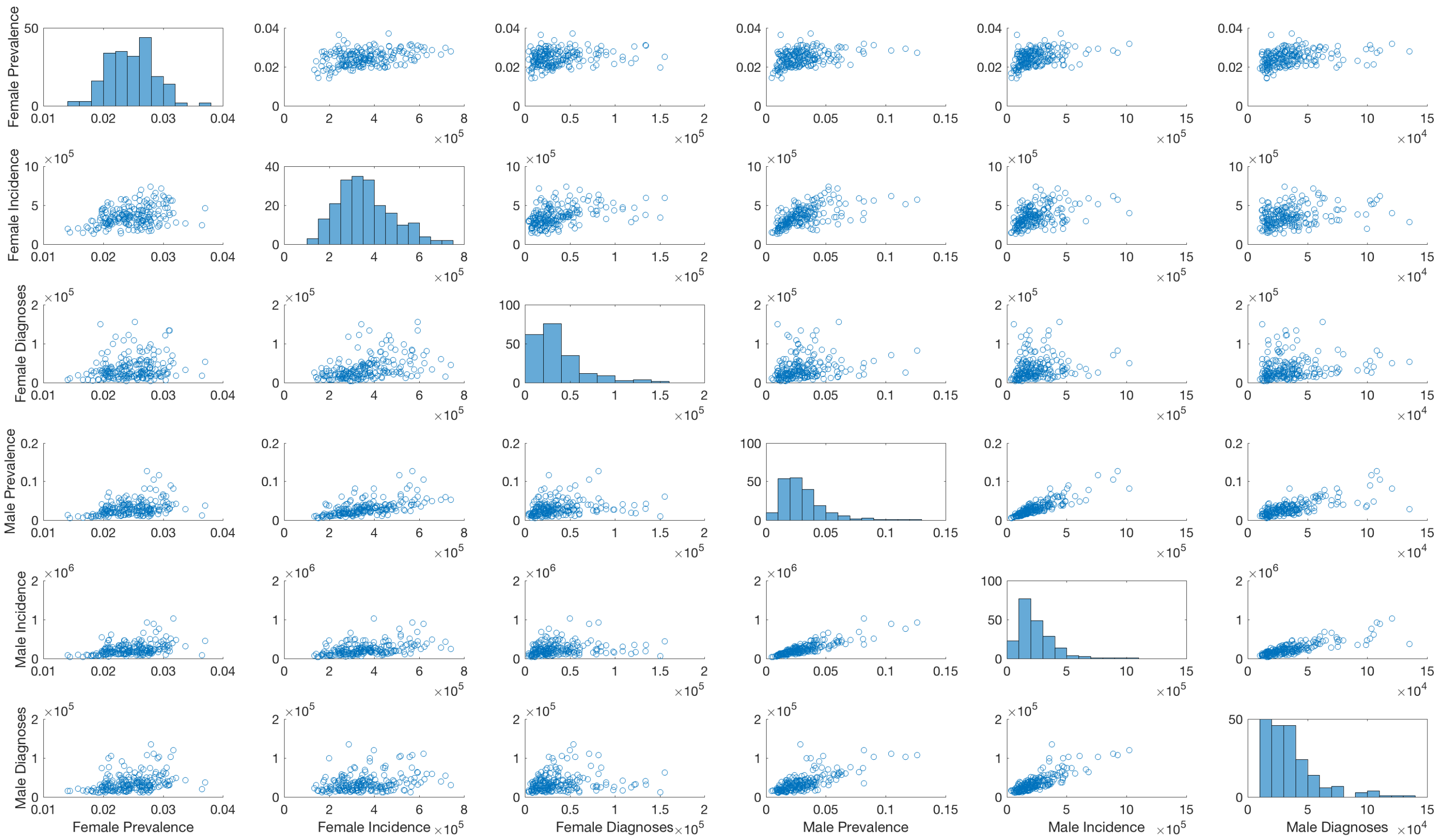

Figure S3: Trellis plots of proportion change in incidence and annual diagnoses in each sex, and PID in women at 20 years after implementing NAAT testing for <u>symptomatic</u> patients.

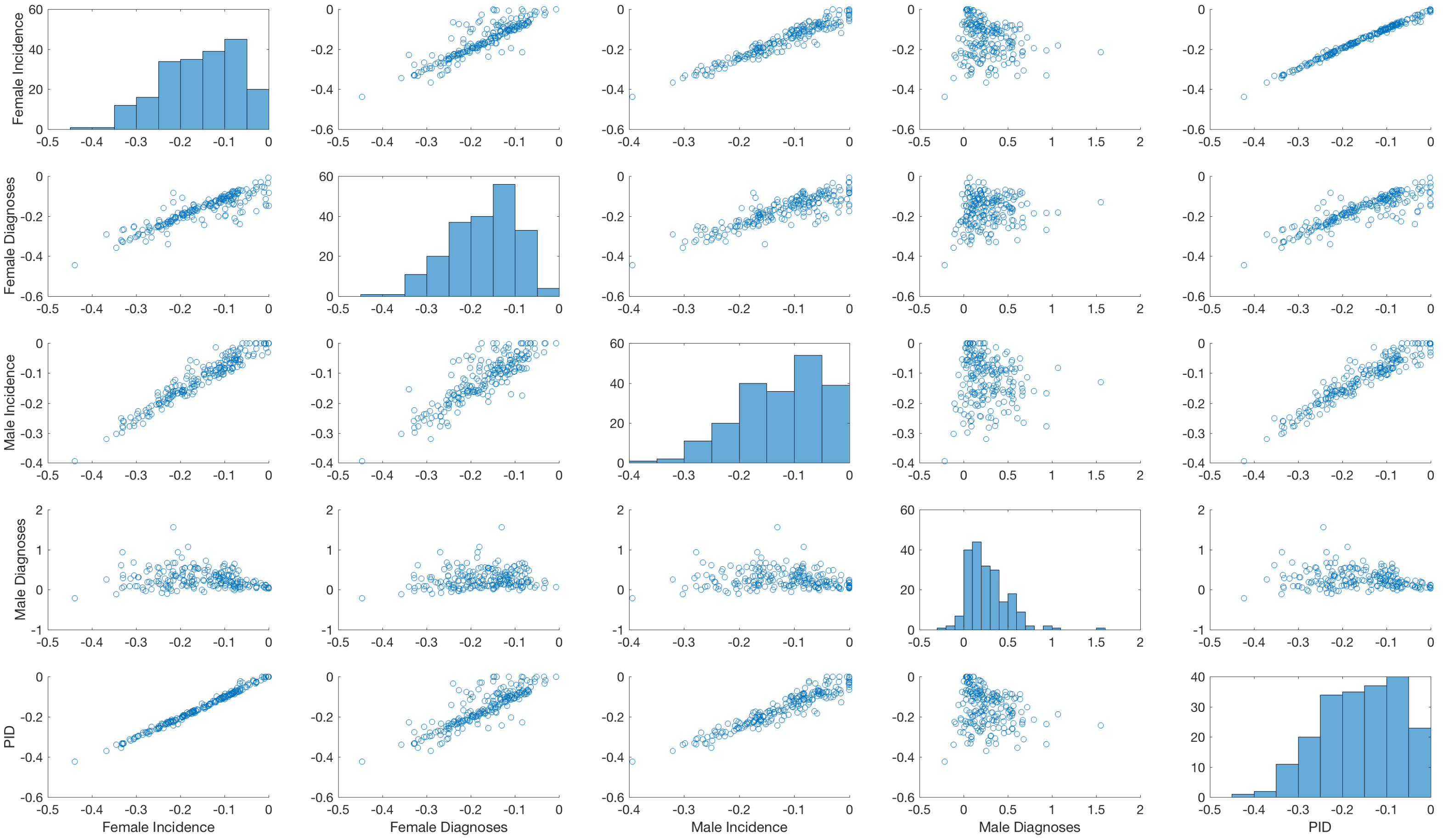

Figure S4: Trellis plots of proportion change in incidence and annual diagnoses in each sex, and PID in women at 20 years after implementing NAAT testing for <u>all</u> patients.

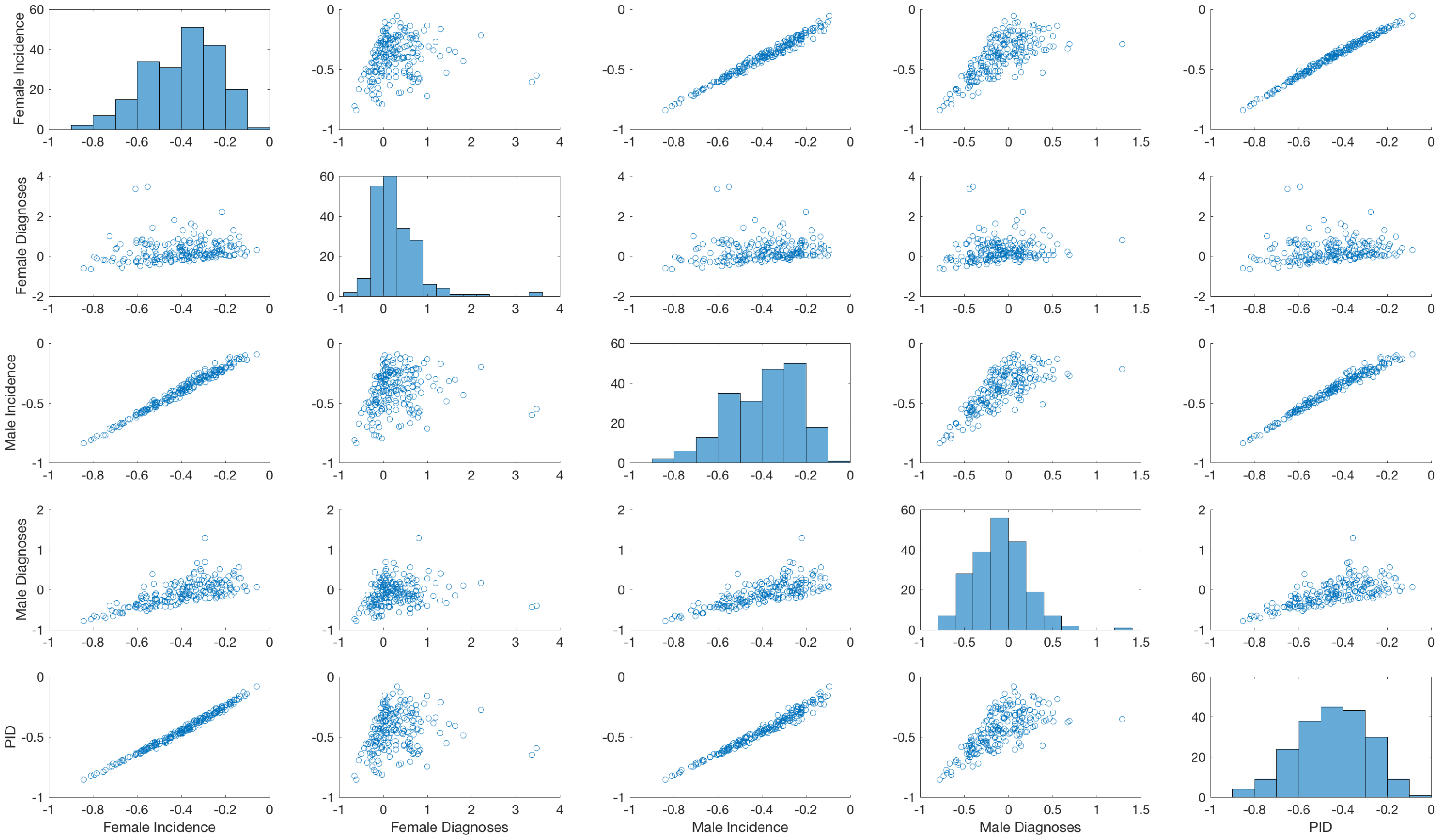

Supplement: Supplementary file 1 — Supplementary Information [file 41598_2017_16302_MOESM1_ESM.pdf]
